# Lateral line placodes of aquatic vertebrates are evolutionarily conserved in mammals

**DOI:** 10.1242/bio.031815

**Published:** 2018-05-30

**Authors:** Stefan Washausen, Wolfgang Knabe

**Affiliations:** Department Prosektur Anatomie, Westfälische Wilhelms-University, 48149 Münster, Germany

**Keywords:** Posterior placodal area, Apoptosis, Lateral line, Vestigial placodes, Neuromasts, Mouse embryos

## Abstract

Placodes are focal thickenings of the surface ectoderm which, together with neural crest, generate the peripheral nervous system of the vertebrate head. Here we examine how, in embryonic mice, apoptosis contributes to the remodelling of the primordial posterior placodal area (PPA) into physically separated otic and epibranchial placodes. Using pharmacological inhibition of apoptosis-associated caspases, we find evidence that apoptosis eliminates hitherto undiscovered rudiments of the lateral line sensory system which, in fish and aquatic amphibia, serves to detect movements, pressure changes or electric fields in the surrounding water. Our results refute the evolutionary theory, valid for more than a century that the whole lateral line was completely lost in amniotes. Instead, those parts of the PPA which, under experimental conditions, escape apoptosis have retained the developmental potential to produce lateral line placodes and the primordia of neuromasts that represent the major functional units of the mechanosensory lateral line system.

## Abstract (precis)

**Summary:** Inhibition of apoptosis in mouse embryos reveals rudiments of the lateral line system, a sensory system common to fish and aquatic amphibia, but hypothesized to be completely lost in amniotes.

## INTRODUCTION

In vertebrate embryos, many developmental processes take place in the presence of cell death (Glücksmann, 1951; Sanders and Wride, 1995). The ‘programmed’ character of such cell death events was confirmed by the formulation of the apoptosis concept (Kerr et al., 1972). In continuation of this ground-breaking concept, several types of programmed cellular demise are currently distinguished with caspase-driven apoptosis being the major type activated during ontogenetic development (Duprez et al., 2009). Yet we still know remarkably little about the exact ontogenetic functions of apoptosis, especially during early embryogenesis. The present study addresses this issue with the example of placode morphogenesis in the posterior placodal area (PPA) of mice.

**Fig. 1.**
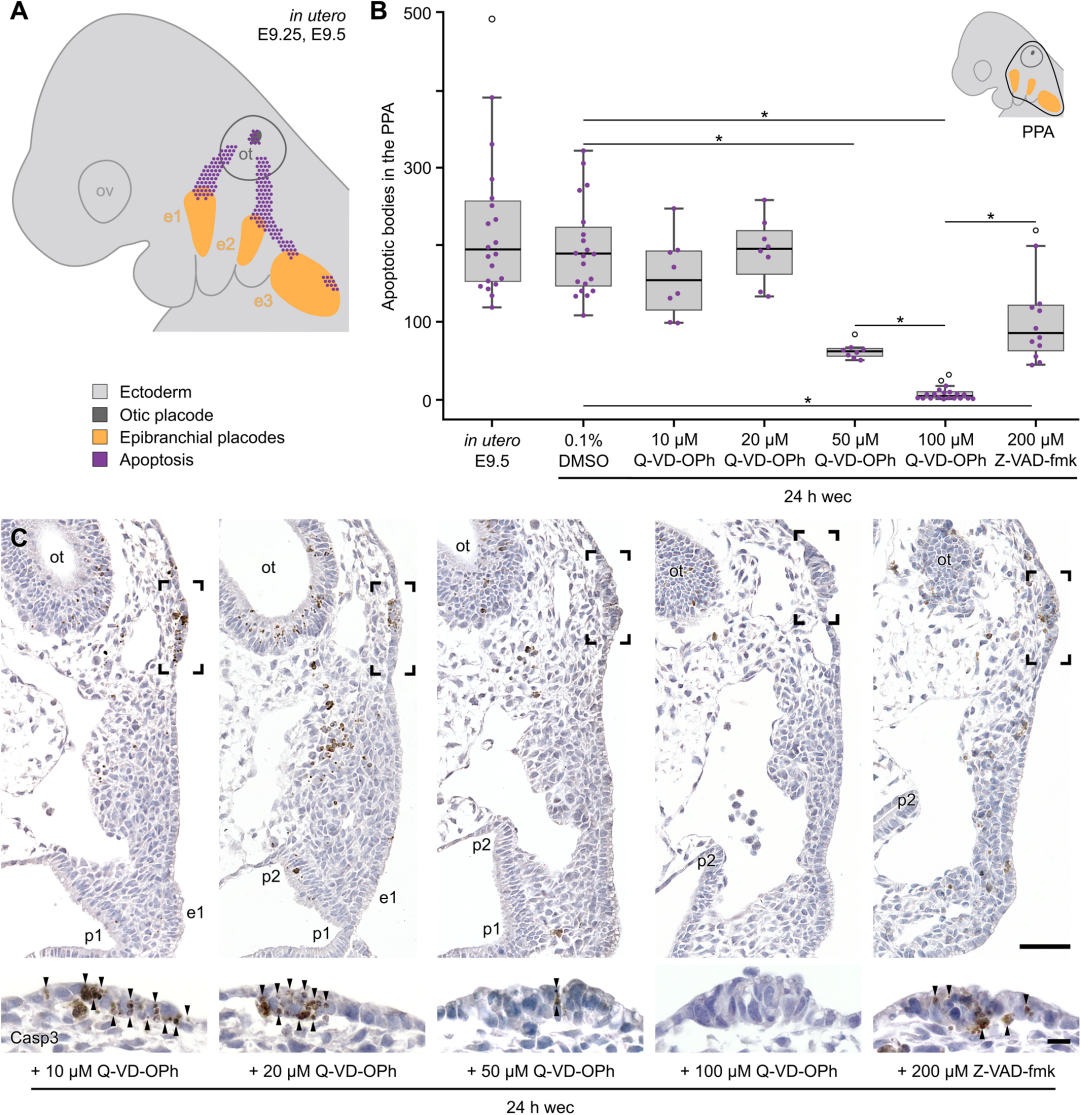
**Pharmacological inhibition of apoptosis in the posterior placodal area (PPA) of embryonic mice.** (A) Summary scheme of *in utero*-developed control embryos including ectoderm (light grey), otic vesicle with detachment site (dark grey), epibranchial placodes (orange), and apoptosis (purple) demonstrates the peak of PPA apoptosis (compiled from Washausen and Knabe, 2013, 2017; *n*=44 body sides). (B) Levels of apoptosis in the PPA (black contour in the schematized embryo; section interval evaluated=10 µm) of *in utero*-developed embryos (*n*=20 body sides) or specimens developed for 24 h in whole embryo culture (wec). Embryos were cultured either in the presence of only the solvent DMSO (control; *n*=20), or in the presence of 10–100 µM of the pan-caspase inhibitor Q-VD-OPh (*n*=8 for 10, 20, or 50 µM, respectively; *n*=20 for 100 µM), or in the presence of 200 µM of the more narrow spectrum caspase inhibitor Z-VAD-fmk (*n*=12). It turned out that Q-VD-OPh treatment reduces PPA apoptosis in a dose-dependent manner. Furthermore, inhibition with 50 µM Q-VD-OPh or 200 µM Z-VAD-fmk is significantly less efficient compared with treatments using 100 µM Q-VD-OPh. Significant differences were measured with unpaired Mann–Whitney test (*P<0.001). Box plots indicate medians (centre lines), 25th and 75th percentiles (box limits), lower and upper extremes (whiskers), data points evaluated separately for each body side (purple dots), and outliers (open circles). (C) Micrographs (standardized sectioning plane) taken from anti-cleaved caspase-3 (Casp3) stained serial sections of mouse embryos treated with 10, 20, 50 or 100 µM Q-VD-OPh or 200 µM Z-VAD-fmk. It turned out that the more effective reduction of PPA apoptosis (arrowheads) is, the better rudiments of the lateral line placodes are preserved. Scale bars: 50 µm (overviews) and 10 µm (magnified insets). E, embryonic day; e1, e2, e3, epibranchial placodes 1, 2, 3, respectively; ot, otic anlage; ov, optic vesicle; p1, p2, pharyngeal pouch 1, 2, respectively.

**Fig. 2.**
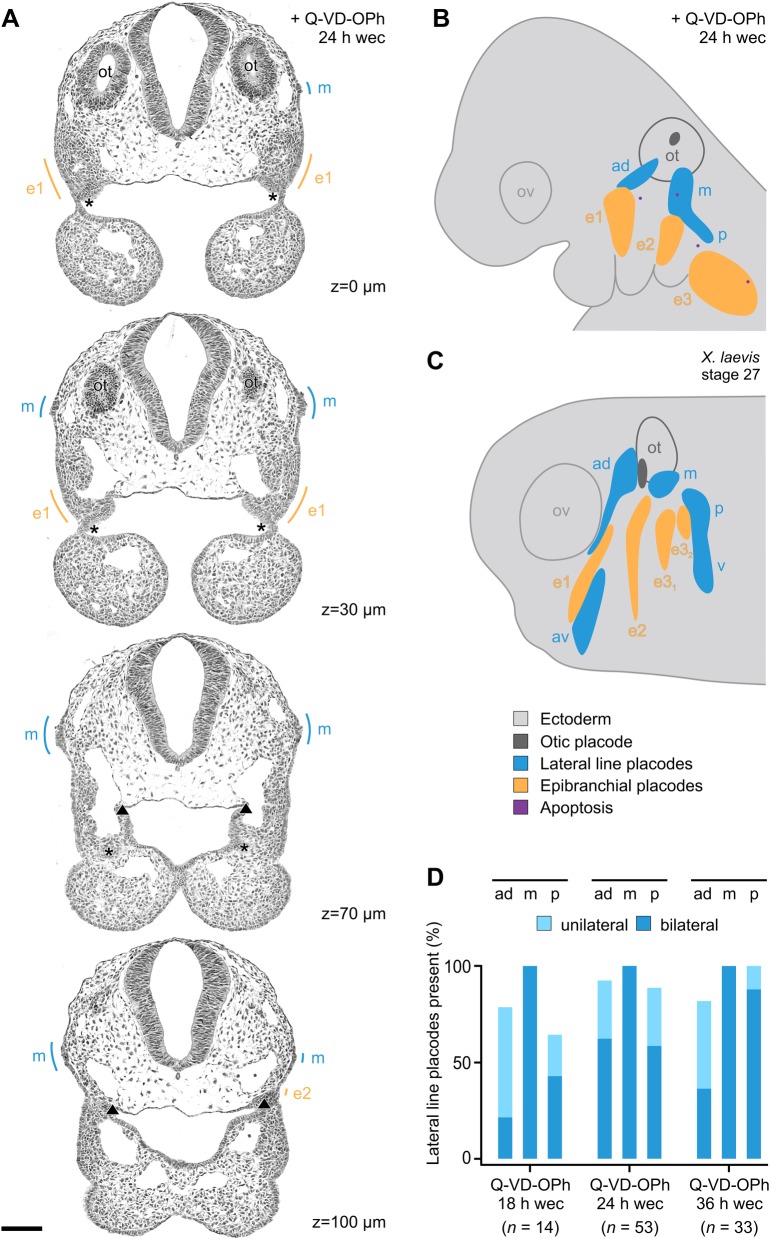
**Pharmacological inhibition of apoptosis generates lateral line placodes in embryonic mice.** (A) Micrographs of representative serial sections used to compile schematic reconstructions of Q-VD-OPh-treated mouse embryos. Exemplarily, topography and dimensions of a middle lateral line placode (m) are shown (embryo #125; relative z-positions indicated). (B,C) Reconstructions of mouse (B) and *Xenopus laevis* (C) embryos show ectoderm (light grey), otic vesicle with detachment site (dark grey), epibranchial placodes (orange), apoptosis (purple), and lateral line placodes (blue). (B) Inhibition of apoptosis for 24 h in whole embryo culture (wec) generates lateral line placodes (embryo #030, mirror-imaged right side). (C) Lateral line placodes in a *Xenopus laevis* embryo, stage 27 (adapted from Schlosser and Northcutt, 2000). (D) Frequency of anterodorsal (ad), middle (m), and posterior (p) lateral line placodes either found unilaterally or bilaterally in the posterior placodal area of mouse embryos exposed to Q-VD-OPh for 18, 24, or 36 h. Scale bar: 100 µm. ad, av, m, p, anterodorsal, anteroventral, middle, and posterior lateral line placode, respectively; asterisk and triangle, pharyngeal pouches 1 and 2, respectively; e1, e2, e3, e3_1_, e3_2_, epibranchial placodes 1, 2, 3, 3_1_, 3_2_, respectively; ot, otic anlage; ov, optic vesicle; Q-VD-OPh, pan-caspase inhibitor; v, migratory primordia of ventral trunk lines.

The multiplacodal PPA is located at the posterior end of the panplacodal primordium and, in amniotes, gives rise to at least three pairs of epibranchial placodes (Fig. 1A,B). These are situated at the dorsal aspects of the branchial arches, and produce visceral sensory neurons of the distal ganglia of cranial nerves VII, IX and X, respectively, that serve roles in the detection of baroreceptive, osmoreceptive or chemoreceptive information from many visceral organs (Baker and Bronner-Fraser, 2001; Baker et al., 2008; Schlosser, 2006, 2010). Other derivatives of the amniote PPA are the otic placode and, in birds, the paratympanic placode which probably provides a barometric or altimetric measurement system (O'Neill et al., 2012; von Bartheld and Giannessi, 2011). The currently held consensus is that only the anamniote PPA is capable of developing lateral line placodes which produce mechanosensory neuromasts or electrosensory ampullary organs unique to aquatic vertebrates (Ghysen and Dambly-Chaudière, 2004; Northcutt, 1992; Schlosser, 2002a, 2006). Three preotic and three post-otic pairs of lateral line placodes represent the primitive condition. However, subsets of lateral line placodes have gone astray, to different degrees, in different gnathostome lineages (Northcutt, 1992, 1997; Schlosser, 2002a, 2006), and their order of ontogenetic appearance reveals interspecies variability (Northcutt and Brändle, 1995; Schlosser and Northcutt, 2000; Fig. 2C).

**Fig. 3.**
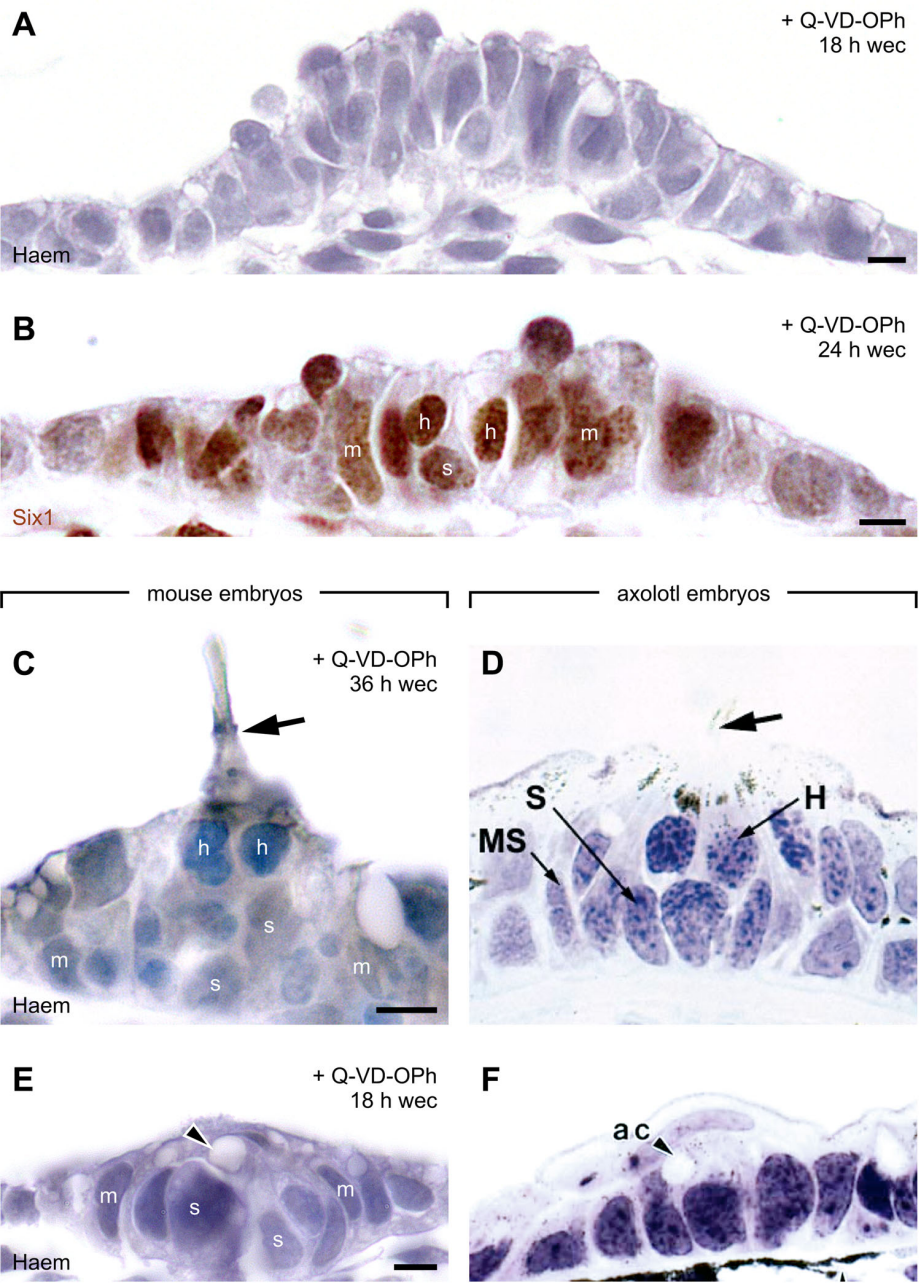
**Lateral line placodes: morphological comparison between Q-VD-OPh-treated mice and axolotl embryos.** (A–C,E) Micrographs taken from serially sectioned mouse embryos. (A) Cubic to columnar, single-row to pseudostratified epithelium of a middle lateral line placode. (B) Six1^+^ middle lateral line placode with neuromast primordium. (C) Neuromast primordium of a posterior lateral line placode with kinocilium (arrow). (E) Neuromast primordium of a middle lateral line placode revealing an apical cavity (arrowhead) that is covered by a flat superficial ectodermal cell. (D,F) Comparison images taken from axolotl embryos (*Ambystoma mexicanum*). (D) Primary neuromast with kinocilium (arrow). Reproduced from Schlosser, 2002b © 2002, with permission from Elsevier. (F) Neuromast primordium with apical cavity (ac; arrowhead) and flat covering cell. Reproduced with permission from Northcutt et al., 1994 © 1994 Wiley-Liss, Inc. All images were adjusted for brightness (including slight gamma changes), colour balance, and sharpness. Scale bars, 5 µm. h, H, hair cell; Haem, haematoxylin staining (Mayer); m, mantle cell; Q-VD-OPh, pan-caspase inhibitor; s, support cell; wec, whole embryo culture; MS, mantle type of supporting cell; S, supporting cell.

During the morphogenesis of epibranchial placodes, embryonic mice demonstrate functionally unexplained apoptosis in three main loci (Washausen and Knabe, 2013, 2017): (1) at the ventral margin of the otic pit [embryonic day (E) 9], (2) between the detachment site of the otic vesicle and the epibranchial placodes 1, 2, and/or 3 (E9.25–E9.5, peak period of apoptosis, Fig. 1A), and (3) in peripheral parts of the three mature epibranchial placodes (E9.5–E11.5). Surprisingly, these apoptotic events predominantly eliminate ectodermal cells that express the general placode marker Six1, a member of the Sine oculis homeobox (Six) family of transcription factors (Washausen and Knabe, 2017). We therefore aimed to determine the developmental potential of these physiologically eliminated placode precursor cells by pharmacologically inhibiting apoptosis in cultured mouse embryos. It turned out that, contrary to previous assumptions, amniotes have retained the capability to produce morphologically and molecularly typical lateral line placodes as well as the primordia of neuromasts. Available evidence suggests that apoptosis may eliminate vestigial lateral line placodes also in other amniotes [Washausen et al. (2005); our unpublished observations in chick embryos]. Our findings further support the hypothesis that lateral line placodes may constitute the default fate of the PPA.

## RESULTS

### Inhibition of apoptosis reveals rudiments of lateral line placodes in mice

In order to learn how apoptosis contributes to placode morphogenesis in the PPA, mouse embryos were removed from the uterus immediately prior to the peak period of apoptosis, and were exposed for 12–36 h in whole embryo culture to the pan-caspase inhibitor Q-VD-OPh (*n*=164). Compared with control embryos (see the Materials and Methods), treatments with 50 µM Q-VD-OPh and, more pronounced, with 100 µM Q-VD-OPh reveal a significant reduction of apoptotic cells in the PPA (Fig. 1). In their places, all embryos treated with 50 or 100 µM Q-VD-OPh (*n*=156) without exception generated morphologically typical lateral line placodes. To demonstrate that formation of these supernumerary placodes depends on the inhibition of caspase-dependent cell death, we performed several additional experiments. First, dose-dependency was demonstrated by treating mouse embryos with 10 or 20 µM Q-VD-OPh. In these cases, significant reductions of apoptotic cells in the PPA and typical lateral line placodes were absent (Fig. 1B,C). Second, other embryos were treated with the more narrow spectrum caspase inhibitor Z-VAD-fmk [less potent caspase-2 and -6 inhibition; Chauvier et al. (2007)]. As expected, application of its common maximum dose [200 µM, whole embryo culture; Massa et al. (2009)] significantly reduces the level of PPA apoptosis and generates supernumerary placodes. However, similar to application of 50 µM Q-VD-OPh, caspase inhibition by 200 µM Z-VAD-fmk is less efficient than inhibition with 100 µM Q-VD-OPh (Fig. 1B,C). Thus, in all further assays, embryos were exposed to 100 µM Q-VD-OPh and, for the purpose of simplification, are further referred to as ‘Q-VD-OPh-treated’ embryos. In Q-VD-OPh-treated embryos, supernumerary lateral line placodes either reside between the detachment site of the otic vesicle and epibranchial placode 1 (anterodorsal lateral line placode) or, respectively, epibranchial placode 2 (middle lateral line placode), and/or immediately posterior to epibranchial placode 2 (posterior lateral line placode) (Fig. 2A,B; Figs S1–S9). Among 100 reconstructed embryos (18, 24, or 36 h in culture), bilateral presence was found in 48% (anterodorsal placode), 100% (middle placode), or 66% (posterior placode) of all cases. At least unilateral development was observed in 87% (anterodorsal placode) or 89% (posterior placode) of all studied embryos (for subgroup analysis, see Fig. 2D).

### Structural properties of lateral line placodes and neuromasts in Q-VD-OPh-treated embryos

**Fig. 4.**
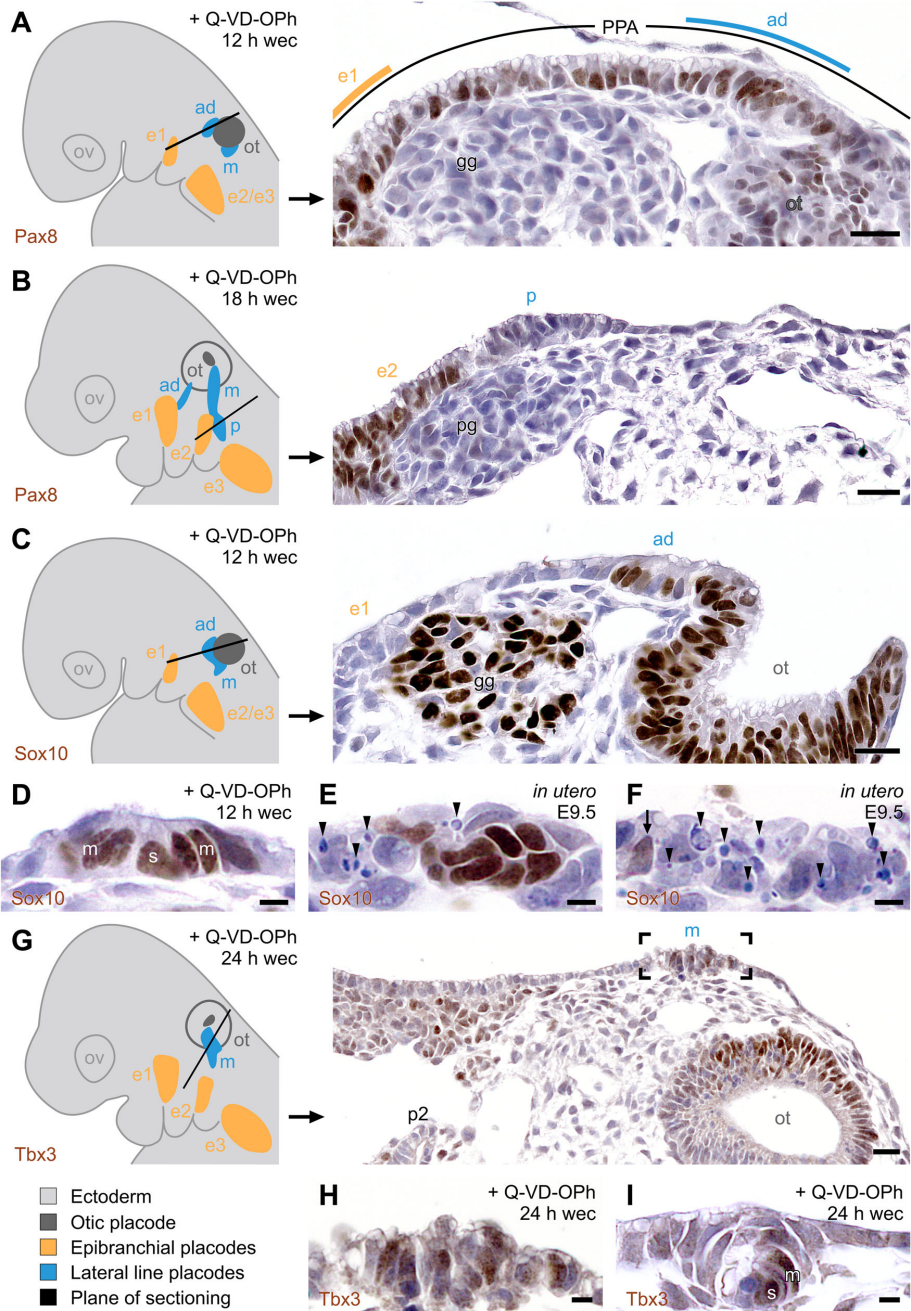
**Lateral line placodes of Q-VD-OPh-treated mice reveal the specific molecular properties of posterior placodes.** (A–C,G) Micrographs taken from serially sectioned mouse embryos, with their positions being shown in the preceding reconstructions that demonstrate ectoderm (light grey), otic pit (A,C) or otic vesicle (B) with detachment site (dark grey), epibranchial placodes (orange), lateral line placodes (blue), plane of sectioning (black line in reconstructions). (A) Pax8 immunopositivity is present in epibranchial placode 1, in the prospective anterodorsal lateral line placode as well as in remaining parts of the thickened PPA. (B) Close apposition of Pax8^+^ epibranchial placode 2 and Pax8^−^ posterior lateral line placode. (C) Anterodorsal lateral line and otic placodes spring from a common Sox10^+^ domain. (D) Neuromast primordium of an anterodorsal lateral line placode with Sox10^+^ mantle (m) and support cells (s). (E,F) During the peak period of PPA apoptosis, *in utero*-developed control embryos [embryonic day 9.5 (E9.5)] demonstrate disorganized Sox10^+^ (E) or predominantly Sox10^−^ ectodermal cells (F**,** but see arrow) as well as high numbers of apoptotic cells (arrowheads) in the positions of vestigial lateral line placodes, here shown for an anterodorsal placode. (G) Scattered Tbx3^+^ cells in a middle lateral line placode (boxed area enlarged in H). (I) Neuromast primordium of an anterodorsal lateral line placode with Tbx3^+^ mantle (m) and support cells (s). Images A–C are stitched from two micrographs using Corel Photo-Paint. All micrographs were adjusted for brightness (including slight gamma changes), colour balance, and sharpness. Scale bars: 20 µm in A–C,G, 5 µm in D–F,H and I. ad, m, p, anterodorsal, middle, and posterior lateral line placode, respectively; e1, e2, e3, epibranchial placodes 1, 2, 3, respectively; gg, geniculate ganglion; ot, otic anlage; ov, optic vesicle; pg, petrosal ganglion; PPA, posterior placodal area; Q-VD-OPh, pan-caspase inhibitor; wec, whole embryo culture.

**Fig. 5.**
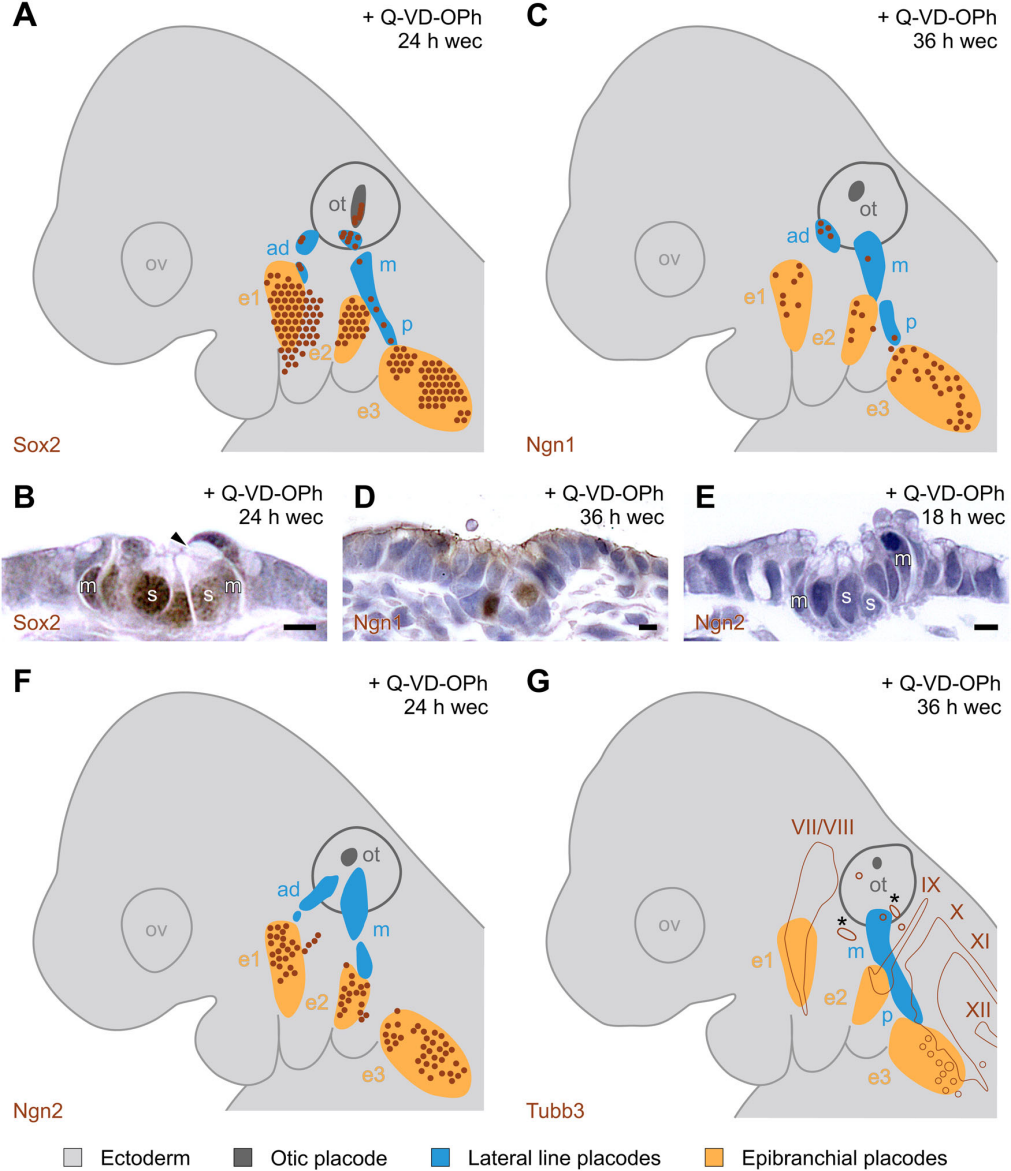
**Neurogenesis in the lateral line placodes of Q-VD-OPh-treated mice.** (A,C,F,G) Reconstructions of serially sectioned mouse embryos (section interval evaluated=10 µm) show ectoderm (light grey), otic vesicle with detachment site (dark grey), epibranchial placodes (orange), lateral line placodes (blue), immunopositive cells (brown dots), developing cranial nerves (partly numbered brown contours in G). (B,D,E) Micrographs taken from serially sectioned mouse embryos. (A) Sox2^+^ cells are present in epibranchial placodes and, to a much lesser extent, in lateral line placodes (embryo #021). (B) Sox2^+^ mantle (m) and support (s) cells in a neuromast primordium of a middle lateral line placode. Note the apical cavity (arrowhead). (C) Ngn1^+^ cells are present in epibranchial placodes and, to a much lesser extent, in lateral line placodes (embryo #039, mirror-imaged right side). (D) Ngn1^+^ neuroblasts in an anterodorsal lateral line placode. (E,F) Ngn2^+^ neuroblasts are present in epibranchial placodes, but absent from lateral line placodes (embryo #001). (G) Tubb3^+^ neurons constitute the anlagen of the cranial nerves 7 and 8 (VII/VIII), 9 (IX), 10 (X), 11 (XI) and 12 (XII). Tubb3^+^ candidates for vestigial lateralis ganglia (asterisks) reside in close proximity to the middle lateral line placode (embryo #014). All micrographs were adjusted for brightness (including slight gamma changes), colour balance, and sharpness. Scale bars: 5 µm. ad, m, p, anterodorsal, middle, and posterior lateral line placode, respectively; e1, e2, e3, epibranchial placodes 1, 2, 3, respectively; ot, otic anlage; ov, optic vesicle; Q-VD-OPh, pan-caspase inhibitor; wec, whole embryo culture.

Lateral line placodes of Q-VD-OPh-treated mice consist of cubic to columnar, single-row to pseudostratified epithelium (Fig. 3A). In addition, they contain various maturation stages of neuromast primordia (Northcutt, 1992; Northcutt et al., 1994; Sato, 1976; Schlosser, 2002b; Stone, 1933; Winklbauer, 1989). Low to moderately differentiated neuromast primordia were identified by their rosette-like arrangement of typically no more than 5–10 epithelial cells per section. Additionally, these forms may reveal pit-like depressions and/or mantle cells with crescent-shaped nuclei that ensheath centrally located, more spherical cells (Figs 3E, 4D, 5B,D,F; Fig. S10A,D). More complex forms (approximately 10­–20 epithelial cells per section) also demonstrate peripherally located mantle cells but, in their centres, allow discrimination between support cells and spherical hair cell precursors placed on top of the support cells (Fig. 3B–D, also for a comparison with axolotl embryos). Furthermore, a few hair cell kinocilia were found (Fig. 3C; Figs S4F, S5F, S6L). Among 100 reconstructed embryos, left- and right-sided PPAs revealed a total of 211 or 231 neuromast primordia, respectively. Fifty-three (12%) of these 442 cases represented complex forms, and up to 6 neuromast primordia were counted per PPA.

Neuromast primordia of anamniotes and Q-VD-OPh-treated mice also share three other characteristics. Firstly, anamniote neuromast primordia reveal an apical cavity covered by flat superficial cells that retreat upon the ‘eruption’ of neuromasts to the surface (Northcutt, et al., 1994; Schlosser, 2002b) (Fig. 3F). Q-VD-OPh-treated mice ‘imitate’ this situation in almost every structural detail (Figs 3E and 5B). Secondly, anamniote secondary neuromasts develop from the mantle zones of primary neuromasts (Northcutt, 1997; Schlosser, 2002b). Correspondingly, pairs or triples of adjacent neuromast primordia were observed in Q-VD-OPh-treated mice (Fig. S10A). Thirdly, in the lateral line system of zebrafish, cell proliferation peaks (1) during gastrulation, (2) within individualized placodes or migrating primordia, and (3) among support cells of the sensory organs (Laguerre et al., 2005). Accordingly, in Q-VD-OPh-treated mice, phospho-Histone H3 immunopositive (pHH3^+^) cells are present among neuromast support cells as well as in non-neuromast bearing parts of the lateral line placodes (Fig. S10B).

### Molecular signature of lateral line placodes and neuromasts in Q-VD-OPh-treated embryos

Lateral line placodes of Q-VD-OPh-treated mice also exhibit the molecular signature of PPA derivatives (Schlosser, 2006, 2010). Firstly, they express the general placode marker Six1 (Fig. 3B; Fig. S8I–K). This retrospectively clarifies why apoptosis, seemingly located ‘interplacodally’ between the otic and epibranchial placodes of *in utero*-developed mice (Washausen and Knabe, 2013, 2017) (Fig. 1A), predominantly removed Six1 immunopositive (Six1^+^) cells (Washausen and Knabe, 2017). Secondly, they spring from paired homeobox (Pax) 2 and/or Pax8 expressing parts of the PPA but, under experimental conditions, lose protein expression of these specific posterior placode markers shortly thereafter (Fig. 4A,B). Thirdly, they develop in tandem with the otic placode, as is apparent from their common expression of Sox10 (Schlosser, 2010), a member of the group E Sox (sex determining region Y-box) family of transcription factors. Prior to the peak period of PPA apoptosis, this common expression domain was observed in both control and Q-VD-OPh-treated embryos. Furthermore, Sox10^+^ epithelial rosettes indicate the onset of neuromast formation (Fig. 4C,D). During the peak period of PPA apoptosis, control embryos reveal disorganized Sox10^+^ epithelia, decreased Sox10 immunoreactivity, and massive apoptosis in the positions of ‘dormant’ lateral line placodes (Fig. 4E,F). In contrast, lateral line placodes of Q-VD-OPh-treated mouse embryos escape apoptosis and demonstrate mosaics of viable Sox10^+^/Sox10^−^ cells as well as intact Sox10^+^ neuromast primordia (Fig. S10C,D). Fourthly, some but not all lateral line placodes and/or neuromasts of Q-VD-OPh-treated mouse embryos express mosaics of Tbx3^+^/Tbx3^−^ cells (Fig. 4G–I). This T-box transcription factor is specifically upregulated in the lateral line placodes of anamniotes (Schlosser, 2006, 2010). Furthermore, Q-VD-OPh-treated (Fig. 4G) as well as *in utero*-developed mice (Bollag et al., 1994) express Tbx3 in the otic vesicle.

As a next step, we examined whether the lateral line placodes of Q-VD-OPh-treated mice express Sox2 which maintains the proliferative status and determines the neural fate of placode precursor cells (Schlosser, 2010). In zebrafish, Sox2 immunopositivity was found in neuromast mantle and support cells, with the latter serving as the source for hair cell replacement (Hernández et al., 2007). Correspondingly, neuromast primordia of Q-VD-OPh-treated mice show Sox2^+^ mantle and support cells (Fig. 5A,B). Altogether, Sox2^+^ cells were observed in 36 out of 66 embryos (Figs S1–S6F), and up to 24 Sox2^+^ cells were present in the lateral line placodes of a single individual (Fig. S2A).

### Neurogenesis in lateral line placodes and ganglia of Q-VD-OPh-treated mice

We then sought to determine whether Q-VD-OPh-treated mice produce neuroblasts destined to populate lateralis ganglia. In zebrafish, *Xenopus tropicalis* and *Xenopus laevis* these premigratory neuroblasts express *Neurogenin1* (*Ngn1*) (Andermann et al., 2002; Nieber et al., 2009; Sarrazin et al., 2006), a proneural gene of the basic helix-loop-helix (bHLH) family of transcription factors. Accordingly, Ngn1^+^ neuroblasts were observed in the lateral line placodes of 6 out of 9 Q-VD-OPh-treated mouse embryos (Fig. 5C,D; Fig. S6G–L and data not shown). However, their numbers were extremely low (no more than 8 Ngn1^+^ cells per single individual: Fig. S6K), and Ngn1^+^ lateralis ganglia were not identified by us. Nevertheless, in rare cases, we found groups of up to 5 β-Tubulin-III immunopositive (Tubb3^+^) neurons in the mesenchyme beneath the lateral line placodes that may represent candidates for such rudimentary ganglia (Fig. 5G; Fig. S10E). Murine epibranchial placodes contain both *Ngn1^+^* and *Ngn2^+^* premigratory neuroblasts (Fode et al., 1998), in this again resembling *X. tropicalis* and *X. laevis* (Nieber et al., 2009). Since the lateral line placodes of Q-VD-OPh-treated mice completely lack Ngn2^+^ neuroblasts (*n*=15 out of 15 embryos; Fig. 5E,F; Figs S7 and S8A–C), differential expression patterns of Ngn1 and Ngn2 help to distinguish between closely apposed Ngn1^+^/Ngn2^−^ lateral line placodes and Ngn1^+^/Ngn2^+^ epibranchial placodes (Fig. 5C–F).

In zebrafish, lateral line placodes, neuromasts and ganglia express the bHLH transcription factor NeuroD1 (Neurogenic differentiation 1) downstream to Ngn1. Furthermore, neuromast hair cells co-express NeuroD1 and the proneural gene Atoh1 (Atonal homolog 1) that, in hair cells of the inner ear, is activated by interactions between Sox2, Six1 and its transcriptional co-factor Eya1 (Eyes absent 1) (Ahmed et al., 2012; Andermann et al., 2002; Itoh and Chitnis, 2001; Sarrazin et al., 2006). Lateral line placodes of Q-VD-OPh-treated mice are immunonegative both for NeuroD1 and Atoh1 (Fig. S8D–H) but, like epibranchial placodes, are partly immunopositive for the LIM-homeodomain protein Isl1 (Islet 1: Fig. S10F) which terminates the expression of Ngn1 and, in turn, NeuroD (Sun et al., 2008). Correspondingly, anamniote lateral line and epibranchial placodes express *Isl1* (Park and Saint-Jeannet, 2010).

## DISCUSSION

Glücksmann (1951) distinguished between three categories of ontogenetic degenerations: ‘morphogenetic’ ones which precede shape changes, ‘histogenetic’ ones that control cell numbers, and ‘phylogenetic’ ones which eliminate vestigial or larval organs. We here provide evidence that, in normally developing mouse embryos, lateral line placodes represent a new case of ‘vestigial organs’ which, unlike larval organs, undergo apoptosis largely prior to the onset of differentiation and, therefore, are particularly difficult to assess. Pharmacological inhibition of apoptosis in Q-VD-OPh-treated mice not only enables short time protection of these vestigial placodes but, far beyond that, allows development of distinct, morphologically and molecularly typical lateral line placodes which even produce neuromast primordia with ciliated hair cells. In this sense, our discovery refutes the long-held evolutionary theory that the whole lateral line sensory system was completely lost in amniotes (Northcutt, 1992; Schlosser, 2002a, 2006).

Our results substantiate, but do not conclusively prove, the hypothesis that lateral line placodes may be considered the default fate of the PPA (Fritzsch et al., 1998; Schlosser, 2010). Originally, this hypothesis had been formulated to explain two observations made in anamniotes: (1) the apparent lack of specific lateral line placode inducers, and (2) the large distance that separates lateral line placodes from known signalling centres in the hindbrain and pharyngeal pouches (Schlosser, 2010). Accordingly, lateral line placodes of Q-VD-OPh-treated mice develop in the absence of externally supplied inducing signals. However, only very recently it was demonstrated that, in zebrafish, anterior lateral line placodes require Fgf signalling, whereas posterior lateral line placodes depend on retinoic acid that inhibits Fgf signalling (Nikaido et al., 2017). Nevertheless, the additional finding that ectopic activation of Fgf or Wnt signalling suppresses posterior lateral line placodes but increases the size of the otic placode is consistent with the hypothesis that (posterior) lateral line placodes may represent the default fate of the PPA (Nikaido et al., 2017).

In view of the fact that culture of E9 mice should not markedly exceed 18–24 h to keep the embryos healthy (Martin and Cockroft, 1999), it is presently impossible to fully explore the developmental potential of vestigial lateral line precursor cells. Thus, for instance, we were unable to determine whether, in embryonic mice, rudiments of the lateral line system can generate migratory primordia, or whether neuromasts principally arise in unmigrated lateral line placodes. Nor can we comment on whether the observed rare occurrence of hair cell kinocilia may be due to culture conditions, or whether these supernumerary placodes normally do not complete the full lateral line differentiation pathway apart from rare exceptions. Both scenarios could explain why we found no evidence for the expression of Atoh1, which is thought to be required for hair cell differentiation (Sarrazin et al., 2006). However, Atoh1 may well have been expressed in the precursors of the few hair cells that differentiated.

Considering that, compared with mice, largely identical patterns of apoptosis were observed in the PPA of the primate-related *Tupaia belangeri* (Tupaiidae, Scandentia, Mammalia) (Washausen et al., 2005) and chick embryos (our unpublished data), apoptotic elimination of vestigial lateral line placodes may prove a widespread phenomenon among amniotes. Our results also shed new light on the possible developmental origin of the mechanosensory paratympanic and spiracular organs (Baker et al., 2008; O'Neill et al., 2012). Recent evidence suggests that the amniote paratympanic and the anamniote spiracular organs are homologous. Furthermore, a Sox2^+^ placode that resides dorsally adjacent to the first epibranchial placode could be identified as the source of the paratympanic organ in chicken embryos (O'Neill et al., 2012). Whether this previously undiscovered placode and, thus, paratympanic and spiracular organs develop independent of both lateral line and epibranchial placodes is not yet fully resolved. Our finding that, in embryonic mice, a latent conservation of mechanisms exists to develop lateral line placodes which are able to recapitulate at least part of the lateral line developmental program increases the probability of a lateral line origin of both organs. Modified versions of our experimental setting may provide an innovative possibility to further explore hitherto unknown developmental links between lateral line, otic and epibranchial placodes (Baker et al., 2008), and the molecular mechanisms that underlie placode morphogenesis in the PPA.

## MATERIALS AND METHODS

### Embryos

All animal handling and procedures were approved by the governmental authorities [Landesamt für Natur, Umwelt und Verbraucherschutz (LANUV), North Rhine-Westphalia, Germany; permit number: 84-02.05.50.16.013]. Timed-pregnant C57BL/6N mice were purchased from Janvier Labs (Le Genest-Saint-Isle, France) and killed by cervical dislocation either 8.5–9 days post-coitum (whole embryo culture) or 9–10.5 days post-coitum (*in utero*-developed control embryos).

### Setting up the whole embryo culture (wec)

For preparation of the culture medium, male Sprague Dawley rat serum (Janvier Labs) was thawed at 37°C, heat-inactivated at 56°C for 30 min, and centrifuged at 2000 ***g*** for 10 min. The resulting supernatant was sterilized through a 0.45 µm syringe filter (723-2545, Thermo Fisher Scientific). After addition of 0.25% antibiotic-antimycotic mix containing 10,000 U/ml penicillin, 10,000 µg/ml streptomycin, and 25 µg/ml amphotericin B (15240096, Thermo Fisher Scientific), the culture medium was transferred to the bottles of the roller culture apparatus (BTC Engineering, Cambridge, UK). For inhibition of apoptosis by covalently blocking the active sites of cysteinyl-aspartate specific proteases (caspases), 100 µM Q-VD-OPh (quinolyl-valyl-O-methylaspartyl-[2,6-difluorophenoxy]-methyl ketone; SML0063, Sigma-Aldrich), a second-generation pan-caspase inhibitor (Caserta et al., 2003; Keoni and Brown, 2015), was dissolved in dimethyl sulphoxide (DMSO; 5179, Carl Roth, Karlsruhe, Germany) and added to the medium (final DMSO concentration: 0.1%). Additionally, a dilution series of Q-VD-OPh (Fig. 1B,C: 10, 20, or 50 µM) was applied to find out whether physiologically occurring apoptosis can be reduced in a dose-dependent manner. For negative controls, (1) Q-VD-OPh was replaced by 100 µM Q-VE-OPh (AG-CP3-0007, AdipoGen, Liestal, Switzerland) where glutamic acid resides in the position of aspartic acid. Alternatively, culture medium was applied (2) with or (3) without 0.1% DMSO. (4) *In utero*-developed embryos served as an additional negative control. For experiments with a more narrow spectrum caspase inhibitor (Fig. 1B,C), Q-VD-OPh was replaced by 200 µM Z-VAD-fmk (carbobenzoxy-valyl-alanyl-aspartyl-[O-methyl]-fluoromethyl ketone; ALX-260-020, Enzo Life Sciences, Lörrach, Germany). Following preparation of the wec apparatus according to the manufacturer's protocol, the bottle content was equilibrated for 1 h at 37.5°C with 40% O_2_, 5% CO_2_, and 55% N_2_ at a gas flow rate of 25 ml/min using a precision gas mixing device (Gmix31, HiTec Zang, Herzogenrath, Germany).

### Whole embryo culture of mouse embryos

The culture was performed according to established protocols (Gray and Ross, 2011; Martin and Cockroft, 1999; New, 1978). The gravid uterus was removed and rinsed in a petri dish filled with Hank's balanced salt solution (HBSS; L2035, Biochrom, Berlin, Germany) at room temperature. Further dissection was performed under a clean bench (HERAGuard HPH 12/95, Thermo Fisher Scientific) using a M165 FC stereomicroscope (Leica, Wetzlar, Germany) and Dumont forceps (11251, 11255, Fine Science Tools, Heidelberg, Germany). The myometrium was torn off in order to expose the decidual swellings that ensheath the embryos. Without damaging the ectoplacental cone, the decidua was peeled from the conceptus and Reichert's membrane was removed from the yolk sac. For staging, each embryo was photographed (microscope camera: DFC450 C, Leica) using the Leica Application Suite (LAS) software, and head length was measured using ImageJ (Rasband, 1997-2016). According to these measurements, somite stages were determined with the help of mouse developmental tables (van Maele-Fabry et al., 1993). Embryos with 9–15 somite pairs [embryonic day (E) E8.5 to less advanced E9] were selected for wec only on the condition that neither their yolk sacs nor their ectoplacental cones had been damaged. Dissected embryos were transferred to the roller culture system using sterile transfer pipettes, and were randomly assigned either to Q-VD-OPh treatment or to one of the above specified control groups 1, 2 or 3 (2–4 embryos per bottle, 1 embryo/ml culture medium). At constant gas supply (40% O_2_, 5% CO_2_, 55% N_2_, gas flow rate: 25 ml/min), embryos were then incubated at 37.5°C and 30 rpm for 12, 18 or 24 h in the dark. For extended incubation periods (30 or 36 h in the dark), gas supply was modified to 70% O_2_, 5% CO_2_, and 25% N_2_ (gas flow rate: 25 ml/min) following 22 h in wec. At the end of culture, embryos were transferred to HBSS (37.5°C) and carefully examined for viability and developmental stage using established morphological criteria and measurements (van Maele-Fabry et al., 1990, 1993). Embryos from all experimental and control groups were selected for further analysis only on the condition that an appropriate developmental status had been reached compared with the corresponding *in utero*-developed embryos.

### Histological procedures

Embryos were removed from the yolk sacs and fixed in 4% paraformaldehyde in phosphate buffered saline (PBS: 137 mM NaCl, 2.7 mM KCl, 10 mM Na_2_HPO_4_, 2 mM KH_2_PO_4_, pH 7.4) for 24 h at room temperature. Following two washes in PBS for 30 min, specimens were dehydrated via an ascending ethanol series. Hereby, embryos were pre-embedded in 1% low gelling-point agarose (Seakem LE, 50001, Lonza, Köln, Germany) at the 50% ethanol level. Following complete dehydration, specimens were cleared in chloroform and subsequently infiltrated as well as embedded in Surgipath Formula ‘R’ paraffin (3801450, Leica). Finally, embryos were serially sectioned at 5 µm using rotary microtomes (RM2245, RM2265, Leica). Serial sections were consecutively placed on two sets of slides (Knabe et al., 2002) that were used either for Mayer's haematoxylin stainings (Romeis, 1948) or for immunostainings with different primary antibodies.

### Antibodies

In the following, each primary antibody is specified along with the corresponding dilution, the incubation parameters applied in our experiments, and with a reference providing details on the antibody specificity. Primary antibodies included: mouse anti-Atonal homolog 1 (Atoh1; Atoh1 supernatant, Developmental Studies Hybridoma Bank, Iowa City, USA, lot 6/20/13, RRID: AB_10805299; 1:50, overnight, 4°C) (Ahmed et al., 2012; Gowan et al., 2001), mouse anti-β-Tubulin-III (Tubb3; clone SDL.3D10, T8660, Sigma-Aldrich, lot 073K4835, RRID: AB_477590; 1:8000, overnight, 4°C) (Miller et al., 2010), rabbit anti-cleaved caspase-3 (9661; Cell Signaling Technology, lot 37, RRID: AB_2341188; 1:8000, overnight, 4°C) (Washausen and Knabe, 2013, 2017), mouse anti-Islet 1 (Isl1; 39.4D5 ascites fluid, Developmental Studies Hybridoma Bank, lot 2/12/09, RRID: AB_2314683; 1:2000, overnight, 4°C) (Karnas et al., 2013), goat anti-Neurogenin1 (Ngn1; sc-19231, Santa Cruz Biotechnology, lot C1215, RRID: AB_2298242; 1:100, 4 h, 37°C) (Krolewski et al., 2013), mouse anti-Neurogenin2 (Ngn2; clone 7G4, MAB3314, R&D Systems, Minneapolis, USA, lot WWI01, RRID: AB_2149520; 1:20,000, overnight, 4°C) (Washausen and Knabe, 2013), goat anti-Neurogenic differentiation 1 (NeuroD1; sc-1084, Santa Cruz Biotechnology, lot A0616, RRID: AB_630922; 1:800, 2 h, 37°C) (Davies, 2007), rabbit anti-Paired homeobox (Pax) 2 (71-6000; Thermo Fisher Scientific, lot 1117672A, RRID: AB_2533990; 1:4000, overnight, 4°C) (Washausen and Knabe, 2017), mouse anti-Pax8 (ACI 438; Biocare Medical, Pacheco, USA, lot 051712, RRID: AB_10922964; 1:100, overnight, 4°C) (Washausen and Knabe, 2017), rabbit anti-phospho-Histone H3 (pHH3; 9701, Cell Signaling Technology, lot 13, RRID: AB_331535; 1:1000, 2 h, 37°C) (Loponen et al., 2011), rabbit anti-Sine oculis homeobox 1 (Six1; HPA001893, Atlas Antibodies, Stockholm, Sweden, lot A00939, RRID: AB_1079991; 1:800, overnight, 4°C) (Washausen and Knabe, 2017), mouse anti-Six1 (clone CLO185, AMAb90544, Atlas Antibodies, lot 02852, RRID: AB_2665581; 1:100, overnight, 4°C) that, compared to rabbit anti-Six1, was raised against the same immunogen and shows identical reactivities (present study), mouse anti-Sex determining region Y-box (Sox) 2 (clone 245610, MAB2018, R&D Systems, lots KGQ0311111, KGQ0315062, RRID: AB_358009; 1:200, overnight, 4°C) (Lathia et al., 2007), goat anti-Sox10 (sc-17342, Santa Cruz Biotechnology, lot L2908, RRID: AB_2195374; 1:800, overnight, 4°C) (Chow et al., 2015), mouse anti-Sox10 (sc-365692, Santa Cruz Biotechnology, lot I0516, RRID: AB_10844002; 1:1600, overnight, 4°C) that, compared to goat anti-Sox10, produced identical staining patterns in mice (present study) and rats (Ševc et al., 2014), and rabbit anti-T-box transcription factor (Tbx) 3 (42-4800, Thermo Fisher Scientific, lot SI256120, RRID: AB_2533526; 1:200, 2 h, 37°C) (Kunasegaran et al., 2014). All biotinylated secondary antibodies were purchased from Vector Laboratories, Burlingame, USA: goat anti-rabbit (BA-1000, RRID: AB_2313606), goat anti-mouse (BA-9200, RRID: AB_2336171), and horse anti-goat (BA-9500, RRID: AB_2336123).

### Immunohistochemistry

Deparaffinised and rehydrated sections were washed in Tris-buffered saline (TBS: 0.05 M Tris, 0.15 M NaCl, pH 7.4). Next, antigen retrieval was performed by high-pressure cooking in citrate buffer (0.01 M, pH 6). Following cooling to room temperature, endogenous peroxidase activity was blocked and sections were permeabilized by incubation with 1% H_2_O_2_ and 0.3% Triton X-100 in TBS for 30 min. Thereafter (and in between all following incubation steps), sections were washed three times in TBS (5 min per wash). For anti-Ngn2 staining, reagents and procedures from the mouse-on-mouse kit (BMK-2002, Vector Laboratories) were used to block the sections and to perform the primary and secondary antibody incubation steps. For all other immunostainings, primary antibodies were diluted in Dako REAL antibody diluent which contains background reducing agents (S202230-2, Agilent Technologies, Waldbronn, Germany). Immunoreacted sections were incubated with the appropriate biotinylated secondary antibody diluted 1:100 in TBS with 2% normal serum of the same species as the secondary antibody (S-1000, S-2000, Vector Laboratories) for 1 h at room temperature. For all immunostaining, sections were finally incubated with the avidin-biotin peroxidase complex (Elite ABC reagent, PK-7100, Vector Laboratories) for 1 h at room temperature. Peroxidase reactions were developed with 0.06% 3,3′-diaminobenzidine (DAB; D5637, Sigma-Aldrich) and 0.007% H_2_O_2_ in Tris-HCl buffer (0.1 M, pH 7.6). Afterwards, sections were thoroughly rinsed in distilled water, counterstained with Mayer's haematoxylin (Romeis, 1948), and, following dehydration and clearance, embedded with DePeX mounting medium (18243, Serva, Heidelberg, Germany). Negative controls were performed by omission of the primary antibody and resulted in the absence of immunolabelling.

### Histological analysis

Histological examinations were carried out for Q-VD-OPh-treated embryos (18 h wec: *n*=29, 24 h wec: *n*=57, 36 h wec: *n*=56) and for the corresponding control specimens (18 h: *n*=5, 24 h: *n*=47, 36 h: *n*=20). For orientation purposes, lower numbers of embryos were additionally examined at 12 h wec (Q-VD-OPh: *n*=9, DMSO: *n*=2) or at 30 h wec (Q-VD-OPh: *n*=1, DMSO: *n*=1). Individual Q-VD-OPh-treated embryos were numbered chronologically (#001 to #152). *In utero*-developed control embryos were taken from the developmental period between E9 and E10.5 (*n*=50). Embryo numbers in the Q-VD-OPh and control groups are consistent with, or considerably exceed, those employed in previous studies which were based on comparable methodologies (Huang et al., 2012; Lassiter et al., 2009; Massa et al., 2009). During all histological examinations, the group allocations were not blinded to the investigators. However, analysis was performed according to predefined, objective criteria, and findings were evaluated independently by the two authors.

Immunohistochemistry with antibodies against the transcription factors Atoh1, Isl1, NeuroD1, Ngn1, Ngn2, Pax2, Pax8, Six1, Sox2, Sox10, and Tbx3 reveals nuclear staining patterns. In cases where immunolabelling was absent from the lateral line anlagen (Atoh1, NeuroD1, Ngn2), internal positive controls were observed as follows: anti-Atoh1 labelling in the dorsal neural tube (alar plate of the hindbrain and spinal cord) (Akazawa et al., 1995), anti-NeuroD1 labelling in premigratory and migrating neuroblasts of the otic vesicle as well as in the cochlear-vestibular ganglion (Davies, 2007; Lawoko-Kerali et al., 2004), and anti-Ngn2 labelling in the epibranchial placodes and in the spinal cord (Fode et al., 1998; Sommer et al., 1996). Other internal positive controls included Isl1 expression in cranial and dorsal root ganglia as well as in developing spinal motor neurons (Zhuang et al., 2013), Ngn1 expression in the trigeminal, epibranchial, and otic placodes as well as in the spinal cord (Fode et al., 1998; Ma et al., 1998; Sommer et al., 1996), Sox2 expression in neural stem cells of the neural tube and otic vesicle (Kiernan et al., 2005), Sox10 expression in neural crest cells (Adameyko et al., 2012), and Tbx3 expression in the ventral pharyngeal endoderm, neural crest-derived mesenchyme, and the otic vesicle (Bollag et al., 1994; Mesbah et al., 2012). To ensure specificity of anti-Pax2, anti-Pax8, and anti-Six1 immunostainings, internal positive and negative controls were applied as previously described (Washausen and Knabe, 2017). Anti-Tubb3 immunohistochemistry reveals neuronal cell bodies, dendrites and axons in the central and peripheral nervous system of E9.5 mouse embryos (Liu et al., 2007). Anti-pHH3 staining detects chromatin condensations of mitotic cells as well as weak chromatin condensations of the late G2 phase and G2/M transition (Loponen et al., 2011).

### Analysis of apoptosis

To verify the diagnosis of apoptotic cells in tissue sections, a multiparametric approach has to be applied (Stadelmann and Lassmann, 2000; Taatjes et al., 2008). Accordingly, previously published patterns of apoptosis in the PPA of C57BL/6N mice (E8.5 to E11.5, *n*=65 embryos) had been determined by using combinations of (1) immunohistochemistry against cleaved caspase-3, (2) the TUNEL (terminal deoxynucleotidyl transferase-mediated dUTP nick end-labelling) method, and structural diagnosis in haematoxylin-stained (3) paraffin or (4) ‘semithin’ (1 µm thick) resin sections (Washausen and Knabe, 2013, 2017). It turned out that the peak period of PPA apoptosis takes place in embryos of approximately 19–27 somite pairs that had been classified either to E9.25 (detaching otic vesicle exhibits a small pore) or E9.5 (a solid stalk connects the closed otic vesicle to the overlying ectoderm) (Washausen and Knabe, 2013, 2017). To provide a baseline for the present experimental work, a summary scheme depicting PPA apoptosis during the peak period was compiled from previously studied embryos (Washausen and Knabe, 2013, 2017) (*n*=44 body sides; Fig. 1A). In the current work, apoptotic cells were identified by immunohistochemistry with antibodies against cleaved caspase-3 as well as by established structural criteria (Häcker, 2000; Sanders and Wride, 1995)*.* Furthermore, representative sections were subjected to TUNEL staining according to the protocol published previously (Washausen and Knabe, 2013)*.*

### Reconstructions of the PPA

Fine-grained schematic reconstructions were performed by transferring histological and/or immunohistochemical data from the PPAs of completely serially sectioned Q-VD-OPh-treated or control embryos to basic schemes of the embryonic head that had been generated in CorelDRAW X4 (Corel, Unterschleißheim, Germany) using scanning electron micrographs and three-dimensional reconstructions of the corresponding embryonic stages as a reference (Tamarin and Boyde, 1977; Verwoerd and van Oostrom, 1979; Washausen et al., 2005). Depending on the embryonic stage and/or the plane of sectioning, 60 up to 240 serial sections (section thickness=5 µm) were evaluated per PPA. Case-dependent, either complete series (section interval=5 µm) or every second section (interval=10 µm) were used to reconstruct the PPA. The plane of sectioning was determined according to the positions of various topographical landmarks (e.g. optic and otic vesicles, branchial membranes). Epibranchial placodes were identified structurally as patches of high-grade thickened, pseudostratified epithelium located adjacent to the branchial membranes (Washausen and Knabe, 2013, 2017). Additionally, the otic anlage and, if present, the otic detachment site were mapped. Diagnosis of lateral line placodes and neuromast primordia was based on the criteria which have been previously established in anamniotes (Northcutt, 1992; Northcutt et al., 1994; Sato, 1976; Schlosser, 2002b; Schlosser and Northcutt, 2000; Stone, 1933; Winklbauer, 1989).

### Photomicrographs

Histological sections were examined under an Axioskop 2 MOT microscope (Carl Zeiss, Göttingen, Germany). Micrographs were captured with an Axiocam HR digital camera (Carl Zeiss) and the KS400 image analysis software (v3.0, Carl Zeiss). Following shading correction in KS400, images were cropped, resized, and adjusted for brightness (including slight gamma changes), colour balance, and sharpness in Corel Photo-Paint X4. All adjustments were applied to the whole image and no specific features within the photographs were modified, removed, or inserted.

### Statistics

Statistical analysis was performed using STATISTICA software (v12.0, StatSoft, Hamburg, Germany). Since the numbers of apoptotic cells in the PPA were not normally distributed (Kolmogorov–Smirnov test) and variances between the three groups were not homogeneous (Levene's test), the non-parametric Mann–Whitney test was used to compare the levels of PPA apoptosis between *in utero*-developed embryos, cultured embryos incubated with DMSO, and cultured embryos treated with Q-VD-OPh or Z-VAD-fmk, respectively (Fig. 1B). All tests were two-sided, and *P* values <0.05 were considered statistically significant. Boxplots of PPA apoptosis were created using STATISTICA software. Diagrams demonstrating the frequency of unilaterally or bilaterally developed lateral line placodes were produced in Microsoft Excel (Fig. 2D).

## Supplementary Material

Supplementary information
