# Supplementary material for: Lateral line placodes of aquatic vertebrates are evolutionarily conserved in mammals
Source: Biol Open. 2018 May 30;7(6):bio031815. doi: 10.1242/bio.031815 (PMC6031350; doi:10.1242/bio.031815)
Supplement: Supplementary information [file biolopen-7-031815-s1.pdf]

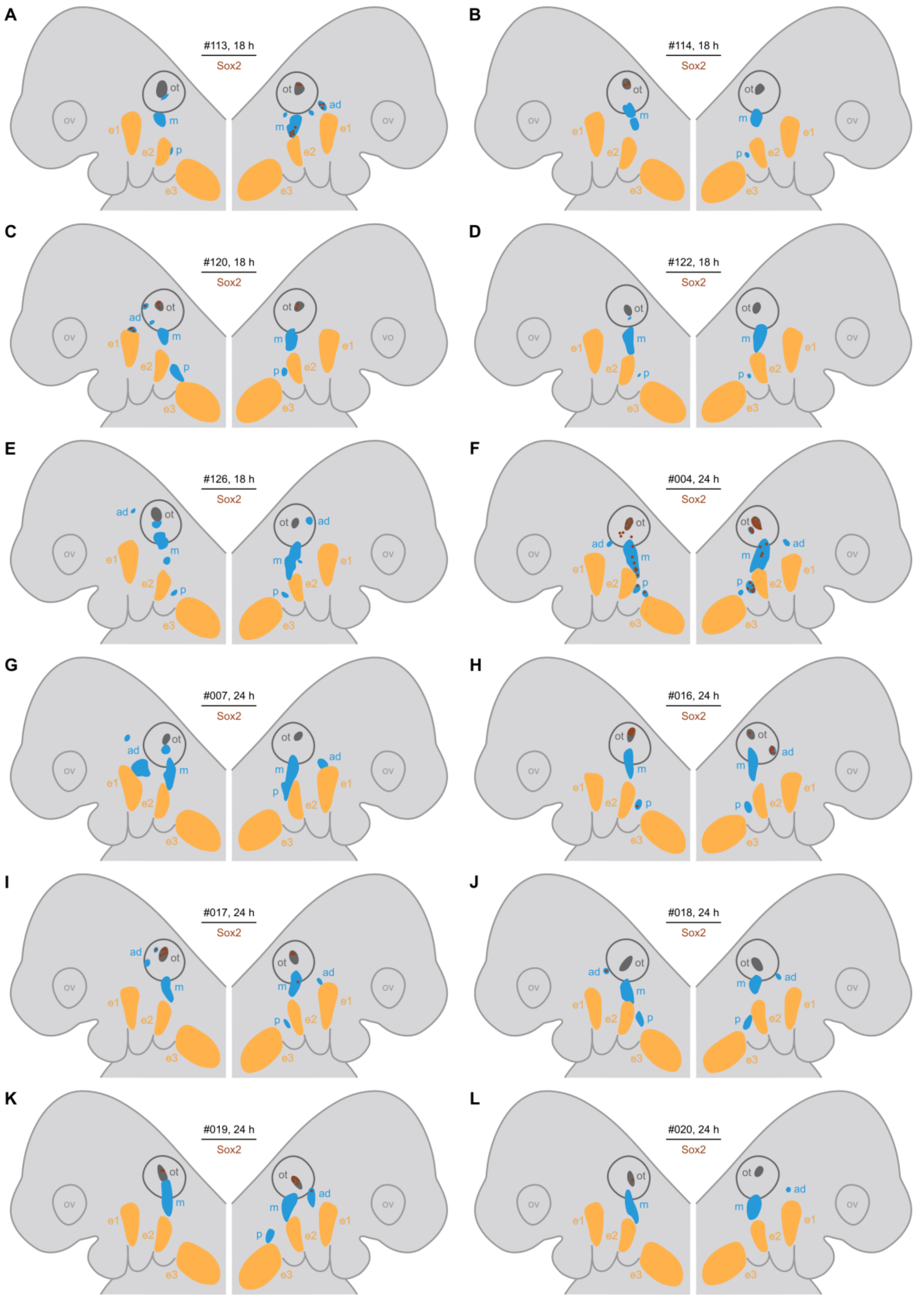

Fig. S1. Pharmacological inhibition of apoptosis generates lateral line placodes in embryonic mice. (A–L) Reconstructions (left and right body sides) of serially sectioned mouse embryos that had been exposed for 18 or 24 h in whole embryo culture to the pan-caspase inhibitor Q-VD-OPh. Paraffin sections (section interval evaluated = 10 µm) were immunoreacted with antibodies against Sox2. Reconstructions show number of embryo in the collection (#), ectoderm (light grey), otic vesicle with detachment site (dark grey), epibranchial placodes (orange), lateral line placodes (blue), immunopositive cells that were exclusively marked in lateral line placodes and at the detachment site of the otic vesicle (brown dots). ad, m, p, anterodorsal, middle, and posterior lateral line placode, respectively; e1, e2, e3, epibranchial placodes 1, 2, 3, respectively; ot, otic anlage; ov, optic vesicle.

Biology Open • Supplementary information

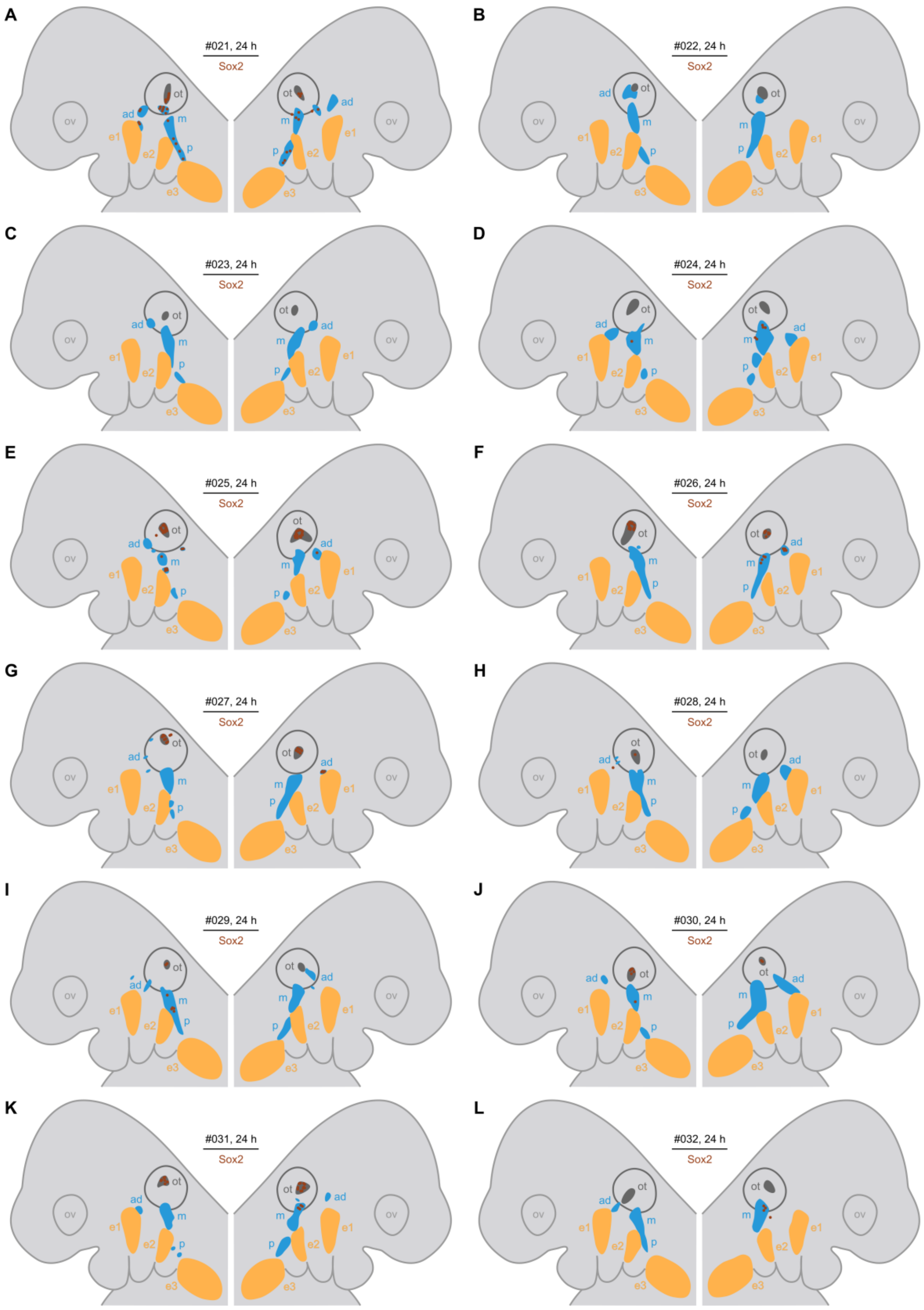

Fig. S2. Pharmacological inhibition of apoptosis generates lateral line placodes in embryonic mice. (A–L) Reconstructions (left and right body sides) of serially sectioned mouse embryos that had been exposed for 24 h in whole embryo culture to the pan-caspase inhibitor Q-VD-OPh. Paraffin sections (section interval evaluated = 10 µm) were immunoreacted with antibodies against Sox2. Reconstructions show number of embryo in the collection (#), ectoderm (light grey), otic vesicle with detachment site (dark grey), epibranchial placodes (orange), lateral line placodes (blue), immunopositive cells that were exclusively marked in lateral line placodes and at the detachment site of the otic vesicle (brown dots). ad, m, p, anterodorsal, middle, and posterior lateral line placode, respectively; e1, e2, e3, epibranchial placodes 1, 2, 3, respectively; ot, otic anlage; ov, optic vesicle.

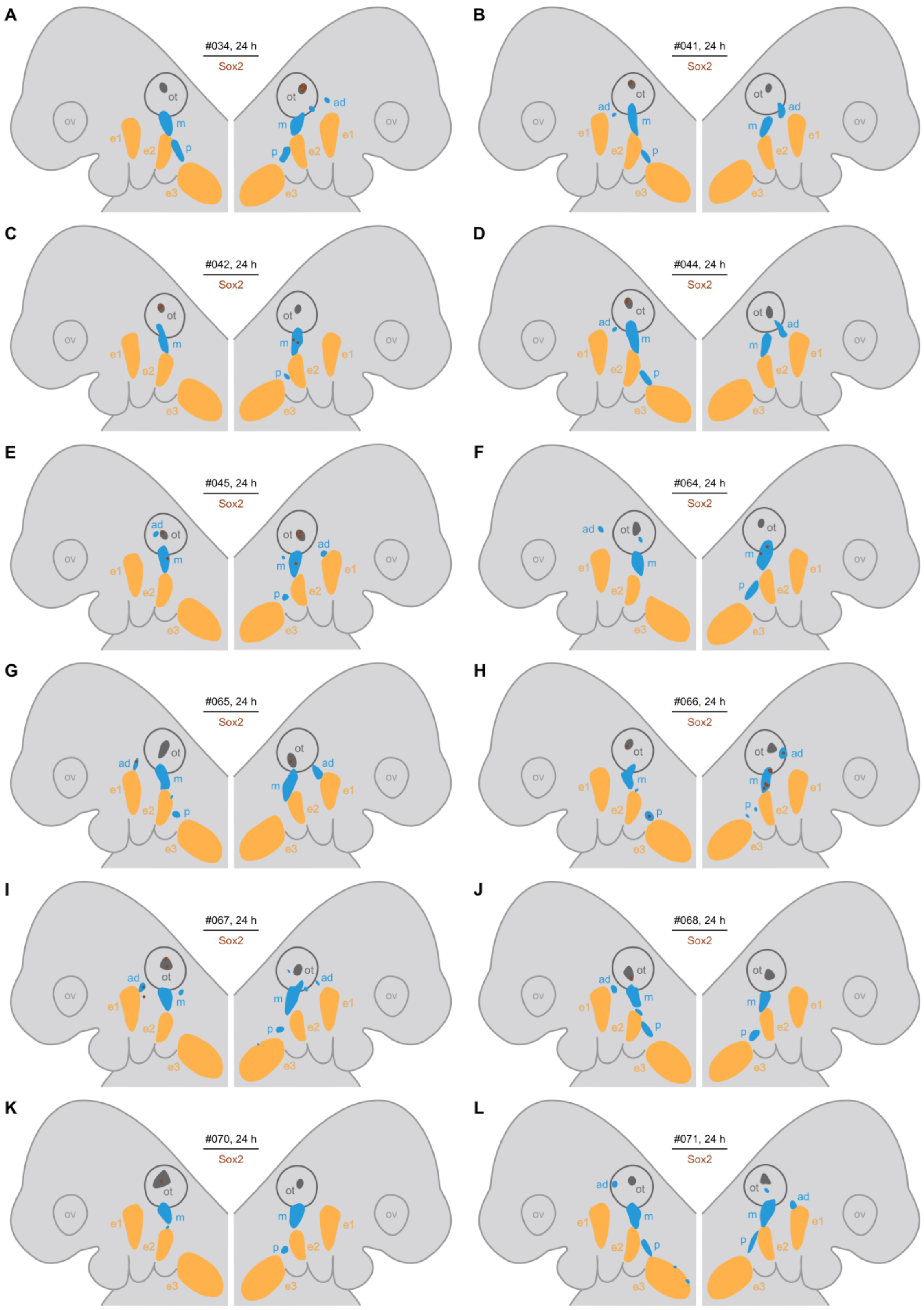

Fig. S3. Pharmacological inhibition of apoptosis generates lateral line placodes in embryonic mice. (A–L) Reconstructions (left and right body sides) of serially sectioned mouse embryos that had been exposed for 24 h in whole embryo culture to the pan-caspase inhibitor Q-VD-OPh. Paraffin sections (section interval evaluated = 10 µm) were immunoreacted with antibodies against Sox2. Reconstructions show number of embryo in the collection (#), ectoderm (light grey), otic vesicle with detachment site (dark grey), epibranchial placodes (orange), lateral line placodes (blue), immunopositive cells that were exclusively marked in lateral line placodes and at the detachment site of the otic vesicle (brown dots). ad, m, p, anterodorsal, middle, and posterior lateral line placode, respectively; e1, e2, e3, epibranchial placodes 1, 2, 3, respectively; ot, otic anlage; ov, optic vesicle.

Biology Open • Supplementary information

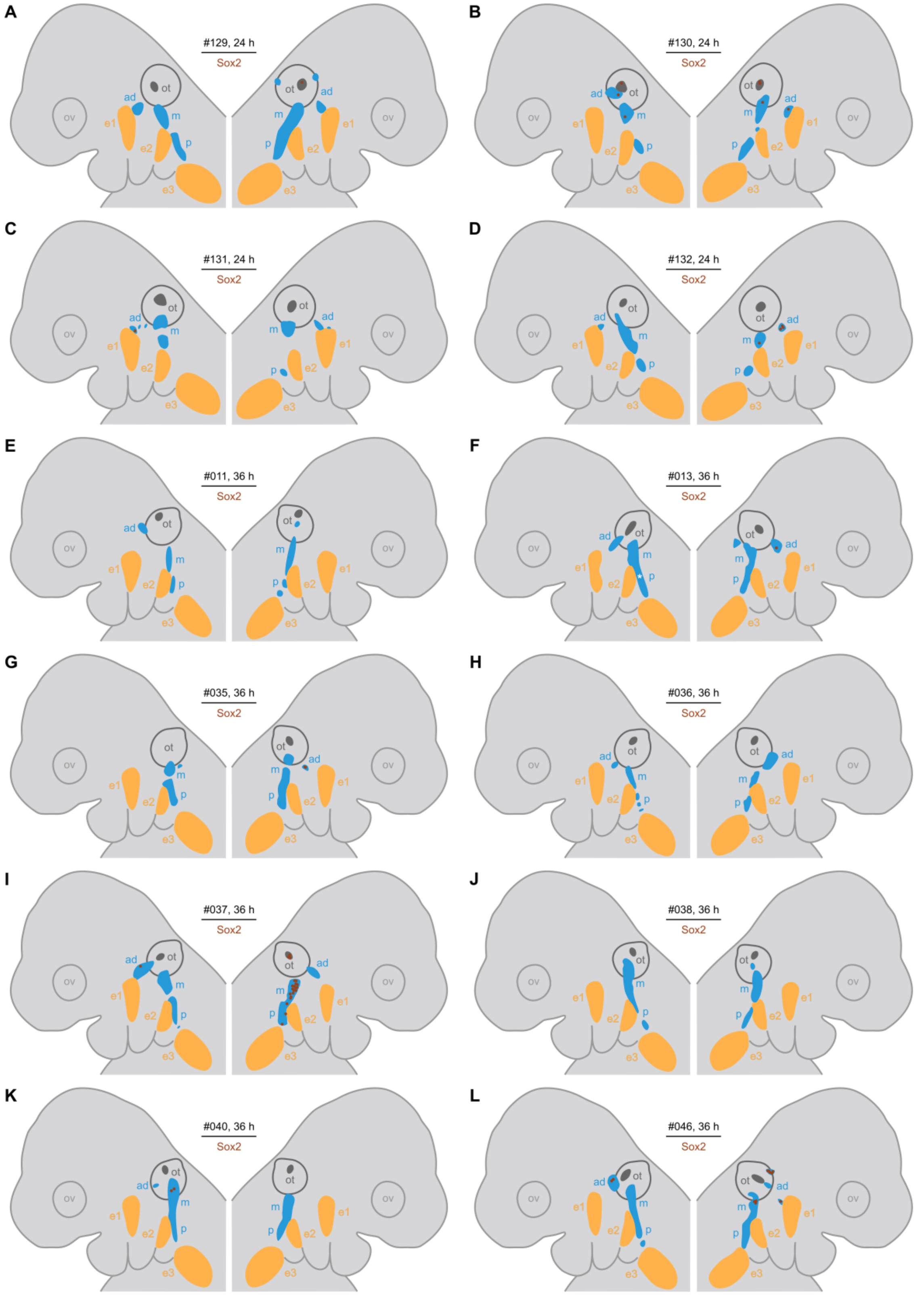

Fig. S4. Pharmacological inhibition of apoptosis generates lateral line placodes in embryonic mice. (A–L) Reconstructions (left and right body sides) of serially sectioned mouse embryos that had been exposed for 24 or 36 h in whole embryo culture to the pan-caspase inhibitor Q-VD-OPh. Paraffin sections (section interval evaluated = 10 µm) were immunoreacted with antibodies against Sox2. Reconstructions show number of embryo in the collection (#), ectoderm (light grey), otic vesicle with detachment site (dark grey), epibranchial placodes (orange), lateral line placodes (blue), immunopositive cells that were exclusively marked in lateral line placodes and at the detachment site of the otic vesicle (brown dots), hair cell kinocilium (white asterisk in F). ad, m, p, anterodorsal, middle, and posterior lateral line placode, respectively; e1, e2, e3, epibranchial placodes 1, 2, 3, respectively; ot, otic anlage; ov, optic vesicle.

Biology Open • Supplementary information

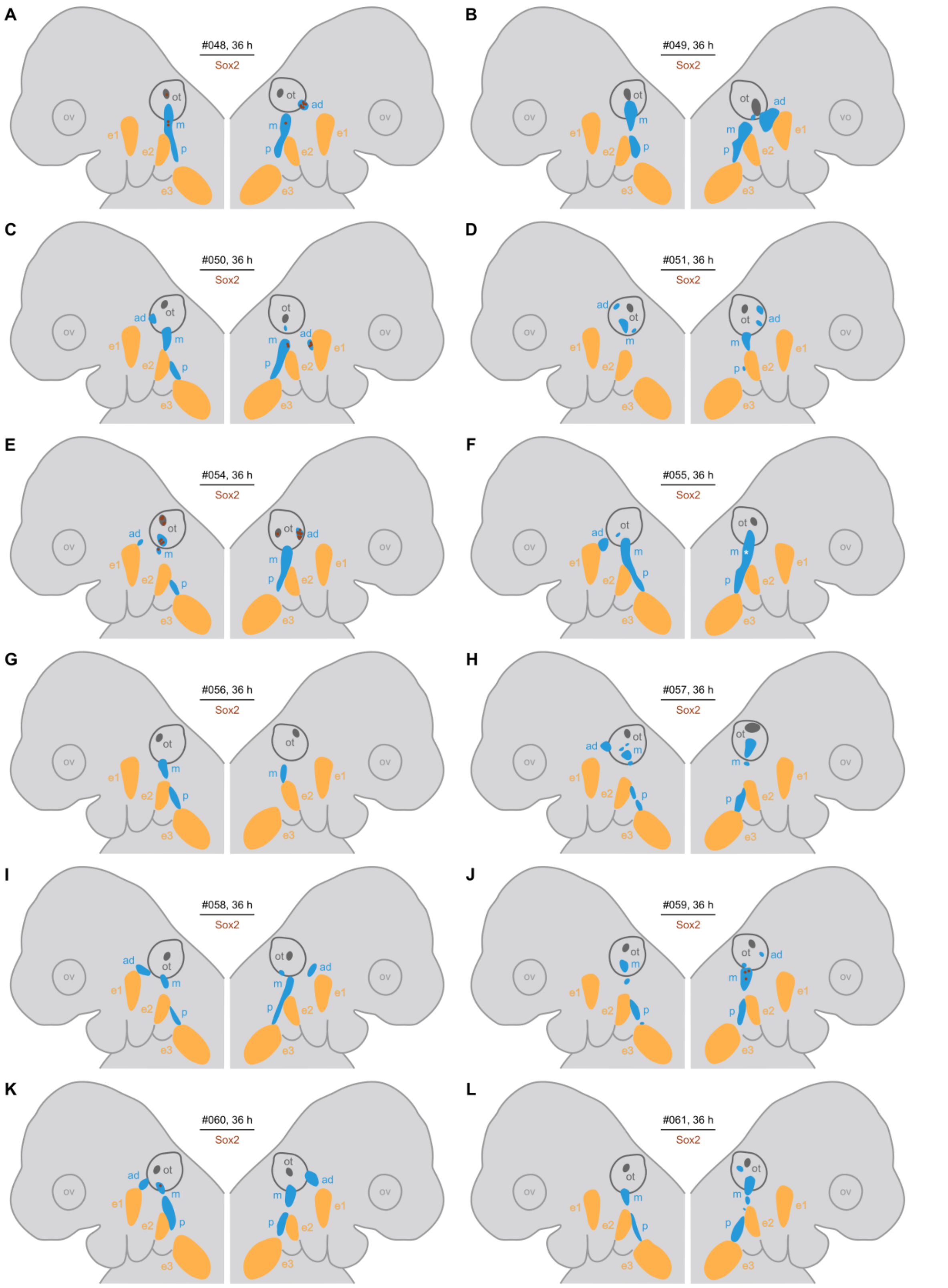

Fig. S5. Pharmacological inhibition of apoptosis generates lateral line placodes in embryonic mice. (A–L) Reconstructions (left and right body sides) of serially sectioned mouse embryos that had been exposed for 36 h in whole embryo culture to the pan-caspase inhibitor Q-VD-OPh. Paraffin sections (section interval evaluated = 10 μm) were immunoreacted with antibodies against Sox2. Reconstructions show number of embryo in the collection (#), ectoderm (light grey), otic vesicle with detachment site (dark grey), epibranchial placodes (orange), lateral line placodes (blue), immunopositive cells that were exclusively marked in lateral line placodes and at the detachment site of the otic vesicle (brown dots), hair cell kinocilium (white asterisk in F). ad, m, p, anterodorsal, middle, and posterior lateral line placode, respectively; e1, e2, e3, epibranchial placodes 1, 2, 3, respectively; ot, otic anlage; ov, optic vesicle.

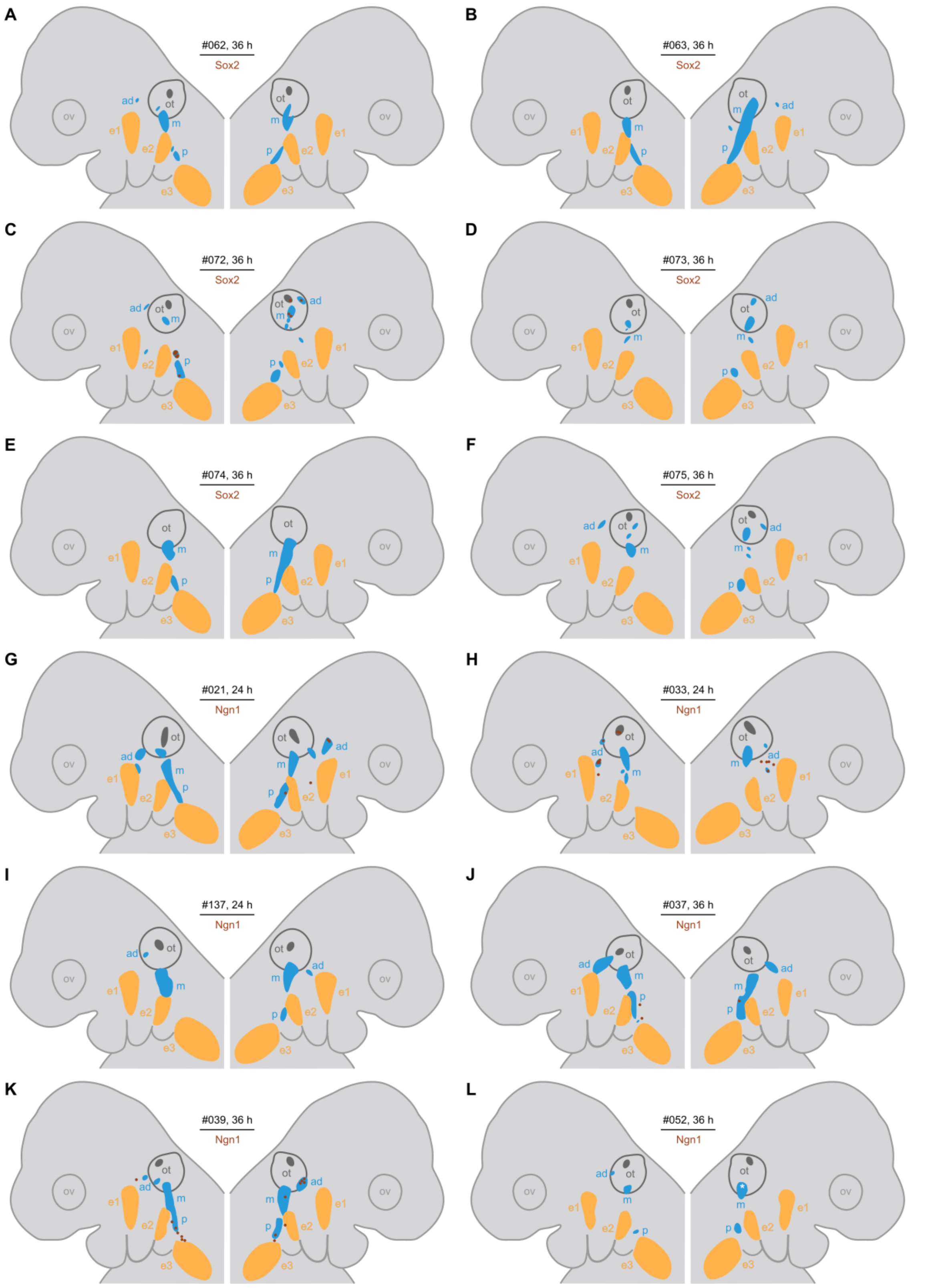

Fig. S6. Pharmacological inhibition of apoptosis generates lateral line placodes in embryonic mice. (A–L) Reconstructions (left and right body sides) of serially sectioned mouse embryos that had been exposed for 24 or 36 h in whole embryo culture to the pan-caspase inhibitor Q-VD-OPh. Paraffin sections (section interval evaluated = 10 µm) were immunoreacted with antibodies against Sox2 (A–F) or Ngn1 (G–L). Reconstructions show number of embryo in the collection (#), ectoderm (light grey), otic vesicle with detachment site (dark grey), epibranchial placodes (orange), lateral line placodes (blue), immunopositive cells that were exclusively marked in lateral line placodes and at the detachment site of the otic vesicle (brown dots), hair cell kinocilium (white asterisk in L). ad, m, p, anterodorsal, middle, and posterior lateral line placode, respectively; e1, e2, e3, epibranchial placodes 1, 2, 3, respectively; ot, otic anlage; ov, optic vesicle.

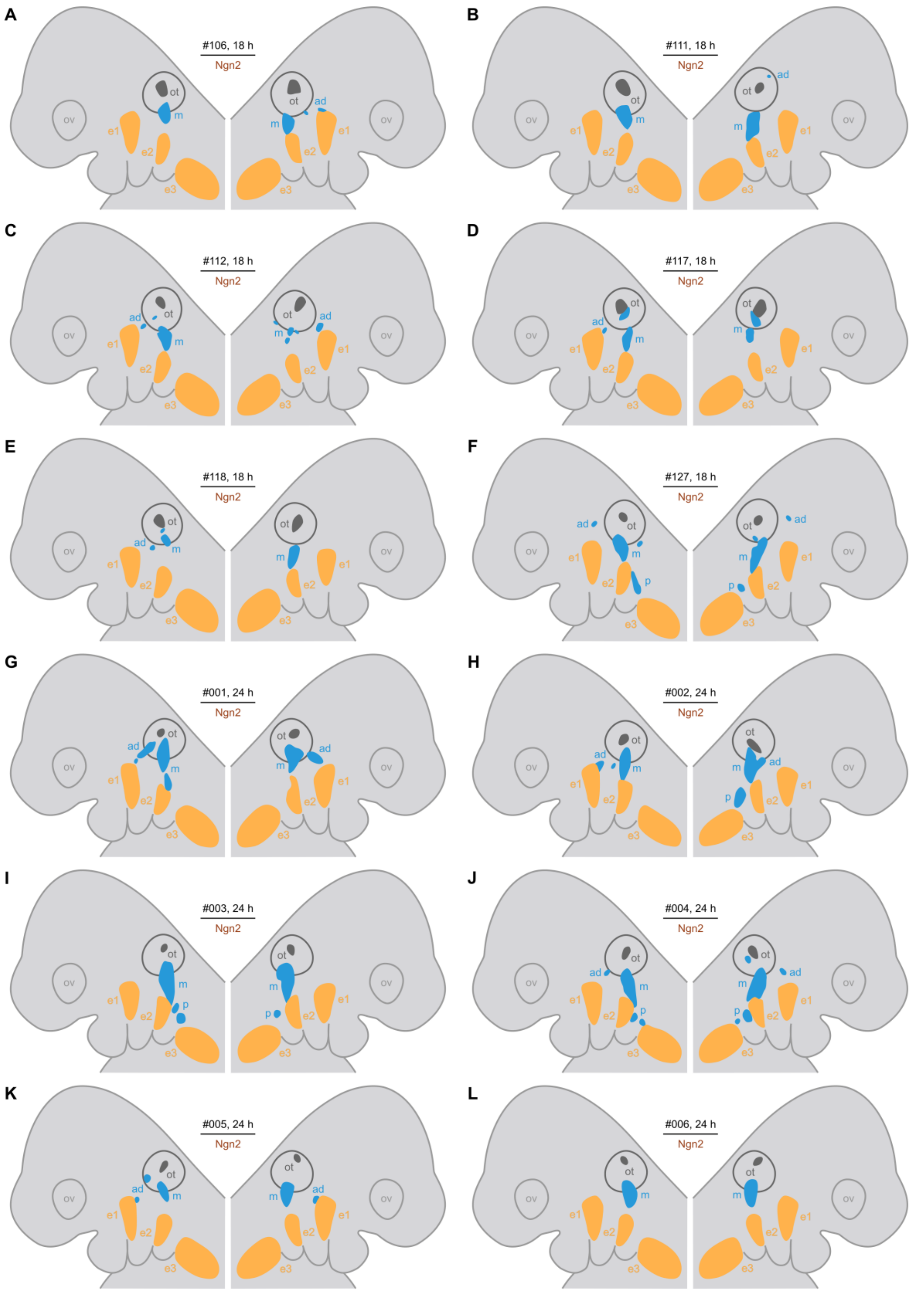

Fig. S7. Pharmacological inhibition of apoptosis generates lateral line placodes in embryonic mice. (A–L) Reconstructions (left and right body sides) of serially sectioned mouse embryos that had been exposed for 18 or 24 h in whole embryo culture to the pan-caspase inhibitor Q-VD-OPh. Paraffin sections (section interval evaluated = 10 µm) were immunoreacted with antibodies against Ngn2. Reconstructions show number of embryo in the collection (#), ectoderm (light grey), otic vesicle with detachment site (dark grey), epibranchial placodes (orange), lateral line placodes (blue), immunopositive cells that were exclusively marked in lateral line placodes and at the detachment site of the otic vesicle (brown dots). ad, m, p, anterodorsal, middle, and posterior lateral line placode, respectively; e1, e2, e3, epibranchial placodes 1, 2, 3, respectively; ot, otic anlage; ov, optic vesicle.

Biology Open • Supplementary information

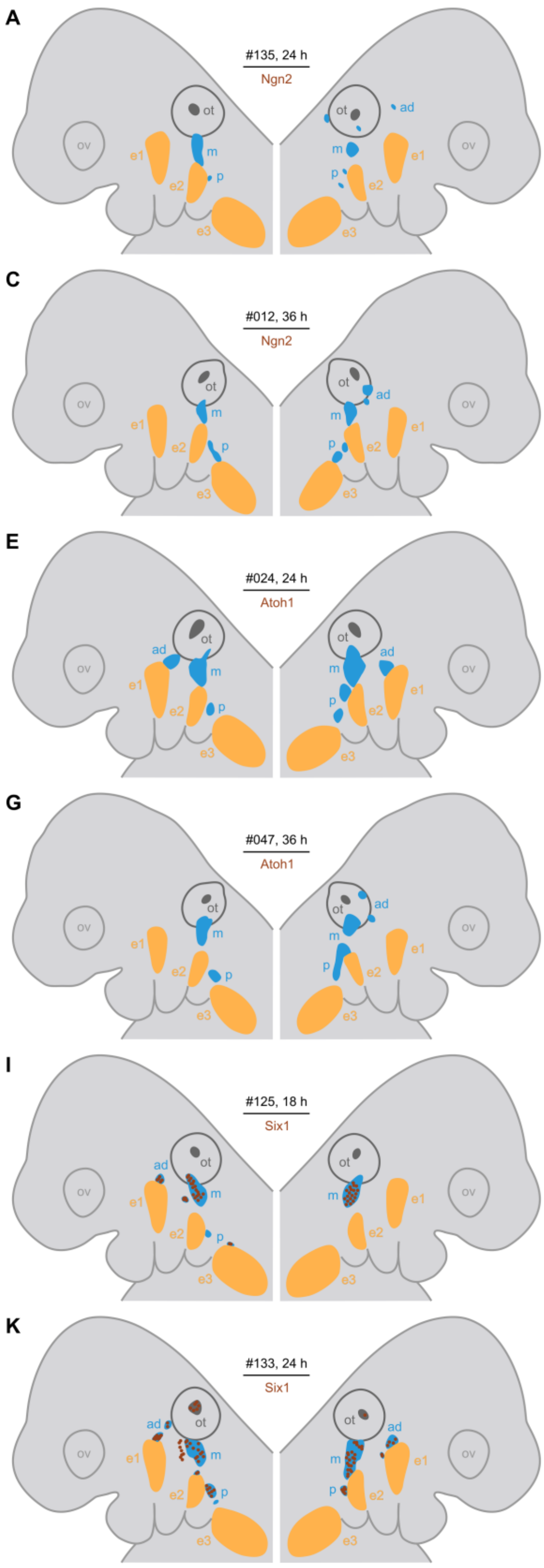

Fig. S8. Pharmacological inhibition of apoptosis generates lateral line placodes in embryonic mice. (A–K) Reconstructions (left and right body sides) of serially sectioned mouse embryos that had been exposed for 18, 24 or 36 h in whole embryo culture to the pan-caspase inhibitor Q-VD-OPh. Paraffin sections (section interval evaluated = 10 µm) were immunoreacted with antibodies against Ngn2 (A–C), Atoh1 (D–H) or Six1 (I–K). Reconstructions show number of embryo in the collection (#), ectoderm (light grey), otic vesicle with detachment site (dark grey), epibranchial placodes (orange), lateral line placodes (blue), immunopositive cells that were exclusively marked in lateral line placodes and at the detachment site of the otic vesicle (brown dots). ad, m, p, anterodorsal, middle, and posterior lateral line placode, respectively; e1, e2, e3, epibranchial placodes 1, 2, 3, respectively; ot, otic anlage; ov, optic vesicle.

Biology Open • Supplementary information

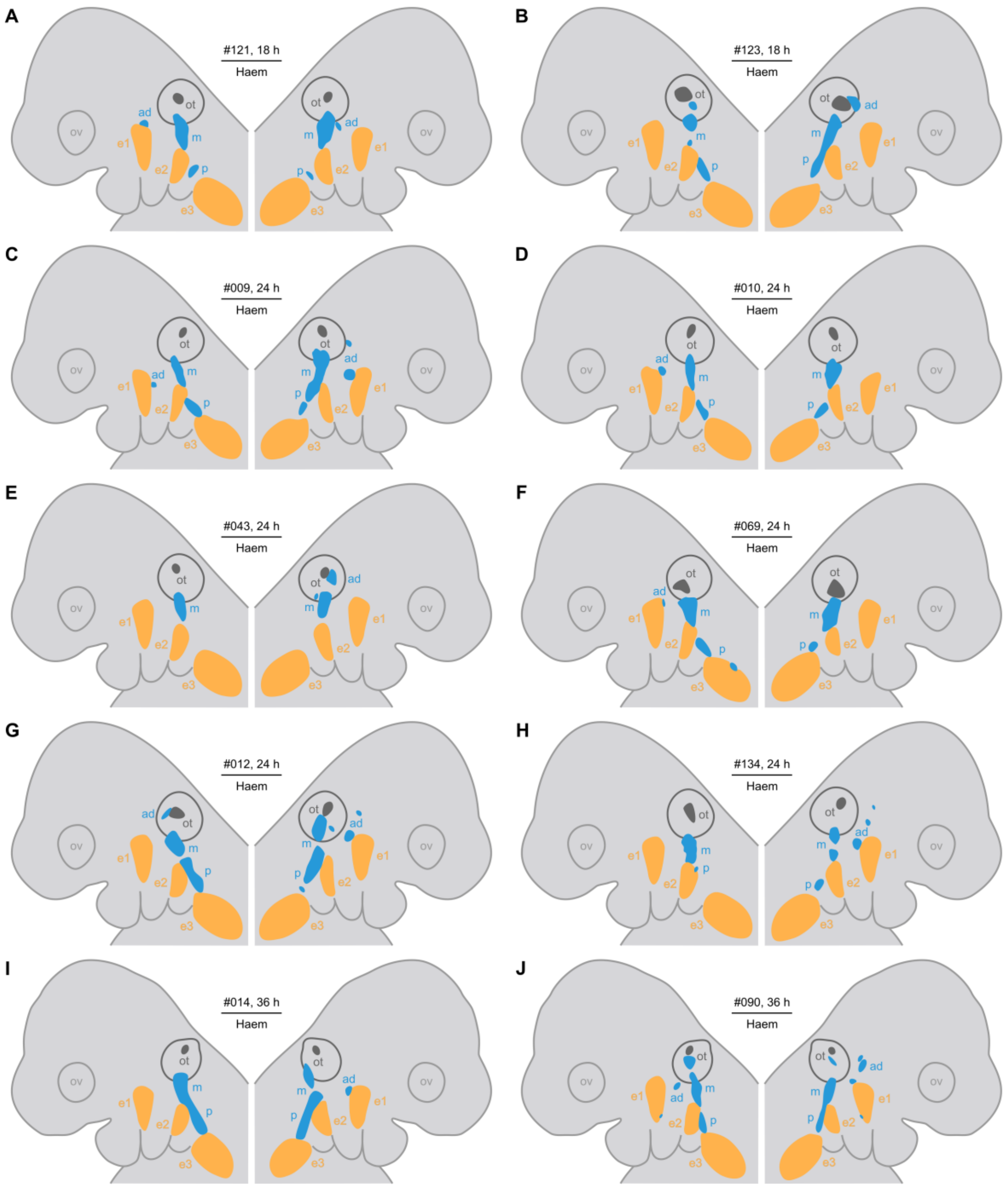

Fig. S9. Pharmacological inhibition of apoptosis generates lateral line placodes in embryonic mice. (A–J) Reconstructions (left and right body sides) of serially sectioned mouse embryos that had been exposed for 18, 24 or 36 h in whole embryo culture to the pan-caspase inhibitor Q-VD-OPh. Paraffin sections (section interval evaluated = 10 µm) were stained with Mayer´s haematoxylin (Haem). Reconstructions show number of embryo in the collection (#), ectoderm (light grey), otic vesicle with detachment site (dark grey), epibranchial placodes (orange), lateral line placodes (blue). ad, m, p, anterodorsal, middle, and posterior lateral line placode, respectively; e1, e2, e3, epibranchial placodes 1, 2, 3, respectively; ot, otic anlage; ov, optic vesicle.

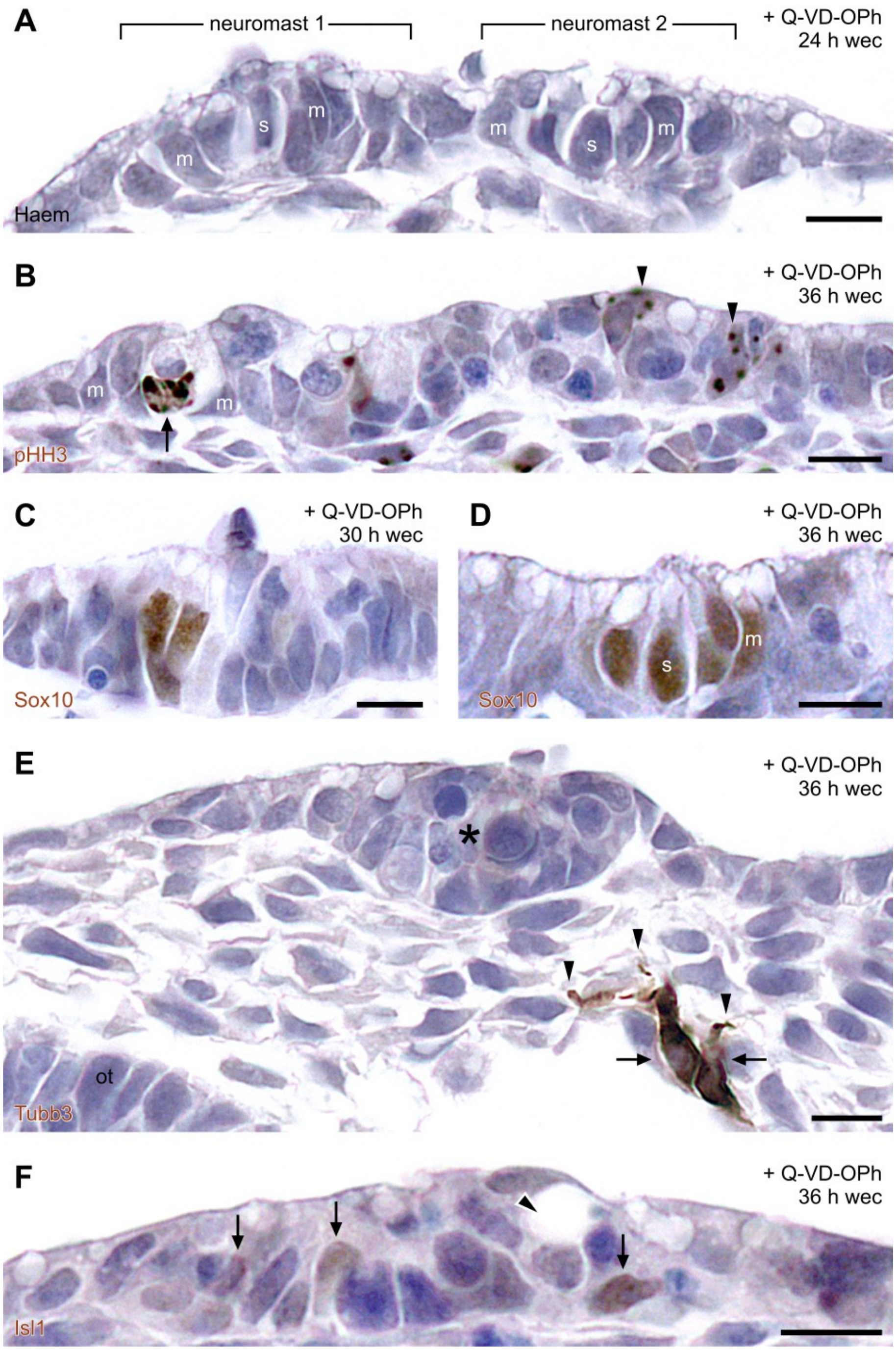

Fig. S10. Structural and molecular properties (pHH3, Sox10, Tubb3, Isl1) of lateral line placodes, neuromast primordia and presumed ganglia in Q-VD-OPh-treated mice. (A–F) Micrographs taken from serially sectioned mouse embryos. (A) Paired neuromast primordia in a middle lateral line placode. (B) Immunohistochemistry with antibodies against pHH3 reveals proliferating cells in a neuromast primordium (support cell indicated by arrow) as well as in non-neuromast bearing parts of a middle lateral line placode (arrowheads). (C,D) Mosaics of viable Sox10^{+}/Sox10^{-} cells and/or intact Sox10^{+} neuromast primorda were found in anterodorsal (C), middle (D), and posterior (not shown) lateral line placodes. (E) Intramesenchymal groups of Tubb3^{+} neurons represent candidates for vestigial lateralis ganglia (arrows) and, here, extend processes (arrowheads) towards a middle lateral line placode (asterisk). (F) Anterodorsal lateral line placode revealing a mosaic of moderately Isl1^{+} (arrows) and Isl1^{-} ectodermal cells. Note the apical cavity (arrowhead) that is covered by a flat superficial cell. Scale bars, 10 µm. Haem, haematoxylin staining (Mayer); m, mantle cell; ot, otic anlage; Q-VD-OPh, pan-caspase inhibitor; s, support cell; wec, whole embryo culture.

Biology Open • Supplementary information
